# Population genetic analysis of the DARC locus (Duffy) reveals adaptation from standing variation associated with malaria resistance in humans

**DOI:** 10.1371/journal.pgen.1006560

**Published:** 2017-03-10

**Authors:** Kimberly F. McManus, Angela M. Taravella, Brenna M. Henn, Carlos D. Bustamante, Martin Sikora, Omar E. Cornejo

**Affiliations:** 1 Department of Biology, Stanford University, Stanford, California, United States of America; 2 Department of Ecology and Evolution, Stony Brook University, Stony Brook, New York, United States of America; 3 Department of Genetics, Stanford University, Stanford, California, United States of America; 4 Centre for Geogenetics, Natural History Museum Denmark, Copenhagen, Denmark; 5 Department of Biological Sciences, Washington State University, Pullman, washington, United States of America; Harvard University, UNITED STATES

## Abstract

The human DARC (Duffy antigen receptor for chemokines) gene encodes a membrane-bound chemokine receptor crucial for the infection of red blood cells by *Plasmodium vivax*, a major causative agent of malaria. Of the three major allelic classes segregating in human populations, the FY*O allele has been shown to protect against *P. vivax* infection and is at near fixation in sub-Saharan Africa, while FY*B and FY*A are common in Europe and Asia, respectively. Due to the combination of strong geographic differentiation and association with malaria resistance, DARC is considered a canonical example of positive selection in humans. Despite this, details of the timing and mode of selection at DARC remain poorly understood. Here, we use sequencing data from over 1,000 individuals in twenty-one human populations, as well as ancient human genomes, to perform a fine-scale investigation of the evolutionary history of DARC. We estimate the time to most recent common ancestor (T_MRCA_) of the most common FY*O haplotype to be 42 kya (95% CI: 34–49 kya). We infer the FY*O null mutation swept to fixation in Africa from standing variation with very low initial frequency (0.1%) and a selection coefficient of 0.043 (95% CI:0.011–0.18), which is among the strongest estimated in the human genome. We estimate the T_MRCA_ of the FY*A mutation in non-Africans to be 57 kya (95% CI: 48–65 kya) and infer that, prior to the sweep of FY*O, all three alleles were segregating in Africa, as highly diverged populations from Asia and ≠Khomani San hunter-gatherers share the same FY*A haplotypes. We test multiple models of admixture that may account for this observation and reject recent Asian or European admixture as the cause.

## Author Summary

Infectious diseases have undoubtedly played an important role in ancient and modern human history. Yet, there are relatively few regions of the genome involved in resistance to pathogens that show a strong selection signal in current genome-wide searches for this kind of signal. We revisit the evolutionary history of a gene associated with resistance to the most common malaria-causing parasite, *Plasmodium vivax*, and show that it is one of regions of the human genome that has been under strongest selective pressure in our evolutionary history (selection coefficient: 4.3%). Our results are consistent with a complex evolutionary history of the locus involving selection on a mutation that was at a very low frequency in the ancestral African population (standing variation) and subsequent differentiation between European, Asian and African populations.

## Introduction

Infectious diseases have played a crucial part in shaping current and past human demography and genetics. Among all infectious diseases affecting humans, malaria has long been recognized as one of the strongest selective pressures in recent human history [1, 2]. The Duffy antigen, also known as DARC (Duffy antigen receptor for chemokines) and more recently as ACKR1 (atypical chemokine receptor 1), is a transmembrane receptor used by *Plasmodium vivax*, a malaria-causing protozoan, to infect red blood cells. *P. vivax* causes a chronic form of malaria and is the most widespread type of malaria outside of Africa [3, 4].

The *DARC* gene has three major allelic types that are the product of two common polymorphisms, forming the basis of the Duffy blood group system [5, 6]. The two variant forms, FY*B and FY*A, are the allelic types commonly observed in non-African populations. FY*B is the ancestral form of the receptor, and is widespread in Europe and parts of Asia. FY*A is defined by a derived non-synonymous mutation (D42G, rs12075) in the *P. vivax* binding region of the DARC protein. It is the most prevalent of the three alleles in modern human populations, with highest frequency in Asia (predicted frequency >80%) and at 30–50% frequency in Europe [4]. FY*A is also present in southern Africa, despite absence from western and central Africa [4, 7–9]. FY*O (also known as Duffy null) is defined by a mutation (T-42C, rs2814778) in the GATA-1 transcription factor binding site in the DARC gene promoter region, and occurs mostly on a FY*B background. The derived FY*O mutation exhibits extreme geographic differentiation, being near fixation in equatorial Africa, but nearly absent from Asia and Europe [4].

Of the three allelic types, FY*A and FY*B are functional proteins, while FY*O does not express the protein on erythrocyte surfaces due to a mutation in the promoter region, which causes erythroid-specific suppression of gene expression [6, 10]. The lack of expression of DARC in erythrocytes has been shown to halt *P. vivax* infection [6, 10]. Moreover, recent evidence shows that heterozygous individuals have reduced DARC gene expression and evidence of partial protection against *P. vivax* [11, 12]. It has been proposed that due to the near-fixation of FY*O, *P. vivax* infection in humans is largely absent from equatorial Africa. An important recent discovery suggests low levels of *P.vivax* infection in FY*O homozygotes [13–17], which indicates that *P. vivax* might be evolving escape variants able to overcome the protective effect of FY*O. Phenotypic effects of the FY*A mutation are less clear than FY*O; however, there is evidence of natural selection and reduced *P. vivax* infection in individuals with this genotype ([18, 19], with conflicting reports in the Brazilian Amazon however [12, 20, 21]).

There is long running interest in characterizing the evolutionary forces that have shaped the Duffy locus. The combination of strong geographic differentiation and a plausible phenotypic association (resistance to malaria) has led to the Duffy antigen being cited as a canonical example of positive selection in the human genome (eg. [22–26]); however, details of its genetic structure remain understudied. Though touted as under positive selection, the few early population genetic studies of this locus found complex signatures of natural selection [27, 28] and it is rarely identified in whole genome selection scans [29–37]. Some genomic loci display signatures of selection readily captured by standard methods, yet other well-known loci, like FY*O, are overlooked potentially due to intricacies not captured by simple models of hard selective sweeps. Detailed analyses of the haplotype structure can help us better understand complicated scenarios shaping genetic variation in loci under selection.

What makes the evolution of FY*O such a complex and uncommon scenario? *Plasmodium* species and mammals have coexisted for millions of years, with frequent cases of host-shifts and host range expansions along their evolution [38, 39]. Great apes are commonly infected with malaria-related parasites [40, 41] and recent evidence suggests that human *P. vivax* originated in African great apes [40], contrasting with previous results that supported an Asian origin for *P. vivax* [42, 43]. In addition to the complex evolutionary relationship among *Plasmodium* species and mammals, the specific mechanisms of invasion of erythrocytes employed by different species are highly diverse and present commonalities among species. *Plasmodium falciparum*, the parasite with the highest prevalence currently in Sub-Saharan Africa presents a highly redundant set of targets that enable erythrocyte invasion, but does not include DARC [44]. On the other hand, DARC erythroid expression influences infection in a variety of other species of *Plasmodium*. For example, it is required for infection by *Plasmodium knowlesi*, a malaria parasite that infects macaques, and SNPs upstream of the *DARC* gene homologue in baboons influence DARC expression and correlate with infection rates of a malaria-like parasite [45, 46].

Despite the general understanding of the relevance of DARC in the evolution of the interaction between *Plasmodium* and primates, a thorough analysis of the complex evolutionary history of this locus using recently available large-scale genomic datasets of diverse human populations is still lacking. Here, we analyze the fine scale population structure of DARC using next-generation sequencing data from twenty-one human populations (eleven African populations), as well as ancient human genomes. We estimate the time to most recent common ancestor of the FY*A and FY*O mutations and estimate the strength of selection on FY*O. We propose a model for the spread of FY*O through Africa that builds on previous findings and provides a more complete picture for the evolution of FY*O. We further explore the relationship between the common FY*A haplotype in Asia and the FY*A haplotype found in southern Africa.

## Results

### Population genetics of the Duffy locus

#### Geographical distribution

**Fig 1:**
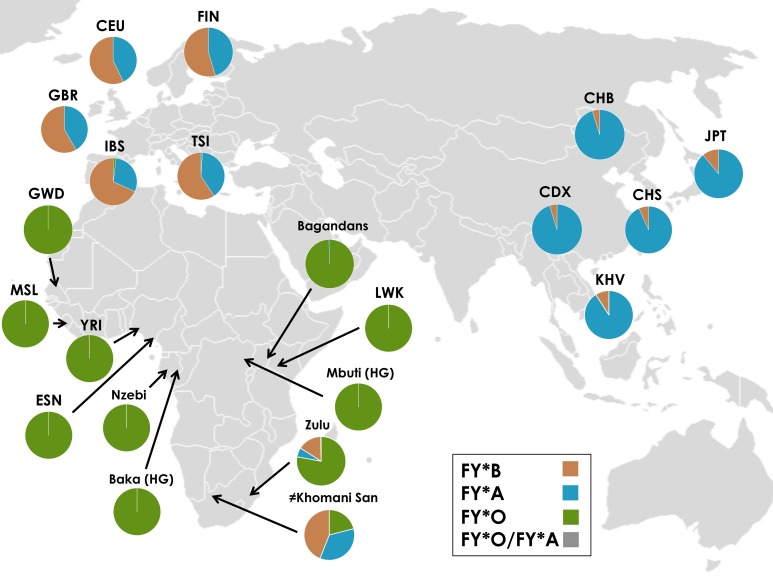
Geographical distribution of allelic classes in samples. *The FY*O/FY*A haplotype is a very rare haplotype that includes both the FY*A and FY*O mutations and is present only in the Zulu samples.

We observe broad consistency between the geographic distribution of the major allelic types in our dataset and previously published results [4] (Fig 1, S2 Table). We find that the FY*O mutation is at or near fixation in western and central African populations, but almost absent from European and Asian samples. All sampled sub-Saharan African populations show frequencies of >99% for FY*O, with the exception of the southern African Zulu and ≠Khomani San populations that contain all three of the FY*A, FY*B and FY*O alleles. FY*A is the dominant allele in all five Asian population samples (89–95%), while FY*B is most common in all five European populations (55–70%).

#### Genealogical relationships

**Fig 2:**
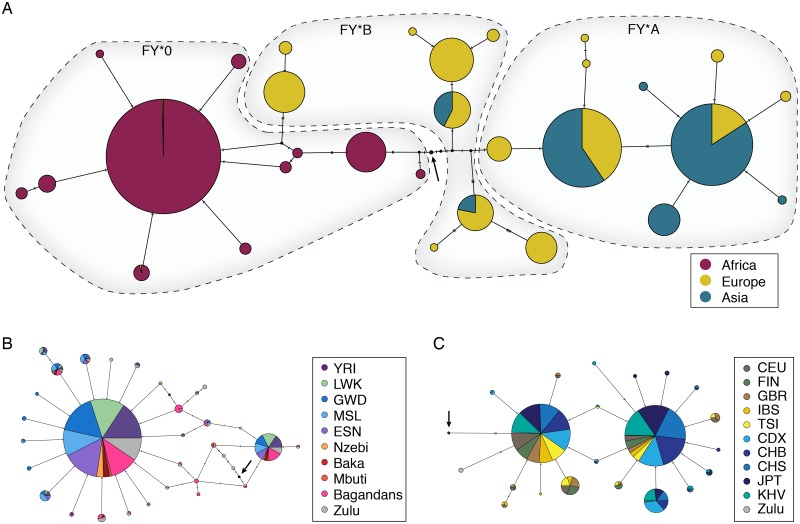
Haplotype networks. Median joining networks of three subsets of haplotypes in the 5kb region centered on the FY*O mutation. The FY*A mutation is located 671 bps downstream from the FY*O mutation. Arrows indicates ancestral sequence. A) All haplotypes observed at least four times B) All FY*O haplotypes observed at least twice. (Note that none of the FY*O haplotypes in this network also carry the FY*A mutation.) C) All FY*A haplotypes observed at least twice. (Note that none of the FY*A hapotypes in this network also include the FY*O mutation.)

We surveyed the 5 kb region surrounding the FY*O mutation. The FY*A mutation is located 671 basepairs downstream of the FY*O mutation. Median-joining haplotype networks of this locus reveal decreased diversity in FY*O and FY*A haplotypes and little geographic structure within continents (Fig 2). We analyzed all haplotypes observed at least four times in this 5kb region and find that FY*O and FY*A allelic classes form distinct clusters, while FY*B is more diverse. Recombination is observed on all haplotypes in this region.

FY*O exhibits two major haplotypes, as seen previously [27], which are defined by four SNPs (chr1:159174095, chr1:159174885, chr1:159176831, chr1:159176856). The haplotypes are at unequal frequency with the most common haplotype at 86% frequency in FY*O sub-Saharan African samples, while the minor haplotype is at 10% frequency. FY*O’s haplotypes exhibit little to no population structure between African populations, though the most common haplotype is at slightly lower frequency in eastern Africa, compared with western and southern Africa. Notably, the FY*O haplotypes observed in the Baka and Mbuti hunter-gatherer populations are identical to Bantu African haplotypes, in stark contrast to the deep divergence between these populations at the genome-wide level [47]. Preliminary analysis of local ancestry around FY*O in the Baka hunter-gatherers shows no increase of Bantu ancestry around FY*O relative to the rest of the chromosome (S7 Fig), indicating the ancient presence of FY*O in both Bantu and hunter-gatherer populations.

The FY*A allele also exhibits two major haplotypes and reduced diversity relative to the ancestral FY*B allele. FY*A’s two common haplotypes segregate at similar frequencies. There is significant recombination between FY*A and FY*B as, unlike FY*O, they coexist in many populations (Fig 1).

#### Distribution in ancient humans

We screened ancient human genomes for the presence of the DARC alleles. We find no evidence for the FY*O mutation, consistent with the absence of genomes from sub-Saharan Africa in currently available ancient DNA datasets. The archaic hominin genomes of the Denisovan and Altai Neandertal carry the ancestral FY*B allele, while an ancient Ethiopian genome dated at 5,000 years old is a FY*A/FY*B heterozygote [48–50]. Additionally, we find that Ust’-Ishim, a 45,000 years old individual from Siberia [51] is also heterozygous for FY*A/FY*B.

Our results confirm the previously observed pattern of extreme geographic differentiation of the DARC region at the continental level. The high F_*ST*_ combined with evidence for resistance to *P. vivax* susceptibility have historically been used as evidence for positive selection at DARC, which is further strengthened by the fact that the FY*O mutation is fixed in a wide variety of highly divergent sub-Saharan African populations. Nevertheless, the presence of two highly diverged FY*O haplotypes indicates the possibility of an ancient origin of FY*O and selection on standing variation, rather than a recent hard sweep. In what follows, we present a series of analyses to test this hypothesis and aim to provide a deeper understanding of the evolutionary history of this locus.

We apply a variety of modern tests of positive selection to re-examine and expand upon previously described signatures of selection at this locus in our expanded population set. We test statistics aimed at detecting both hard and soft sweeps, in order to determine whether these statistics can provide information about the mode or strength of selection in this region. We utilize quantitative measurements to infer if FY*O was a recent selective sweep, similar to many other malaria resistance alleles, or if it occurred in the more distant past. We can use this T_MRCA_ estimate to further characterize selection on FY*O to infer if it is more consistent with a *de novo* mutation or a sweep from standing variation, as well as it’s strength of selection. Lastly, we investigate the ‘equatorial Africa’ portion of our hypothesis. We note the highly diverged southern African San populations carry FY*O at low frequency and we investigate whether this appears to be a recent introgression, supporting our hypothesis of a sweep in equatorial Africa, or if it appears FY*O has been segregating for thousands of years in southern Africa as well.

### Evidence of selection in DARC

#### Evidence of positive selection at FY*O

Despite FY*O’s biological support for positive selection and previous genetic analyses consistent with this hypothesis, it has not been identified as a potential selected region in many genome-wide selection scans [29–37]. Accordingly, we find the DARC promoter region is not an outlier in the genome with respect to segregating sites, average number of pairwise differences nor Tajima’s D (S3 Table). Though the DARC promoter region has the fewest SNPs in African populations, it has more pairwise differences likely due to two divergent FY*O haplotypes in these populations. To further investigate the underlying characteristics that prevents detecting this locus as non-neutral in genome-wide scans of selection, we analyzed statistics from three main classes of selection scans: population differentiation (F_*ST*_), site frequency spectrum (Sweepfinder [52, 53]), and linkage disequilibrium (H-scan [54]) (Table 1, S4–S6 Tables).

**Table 1:** Selection scan results.

	20KB		100KB	
	Sweepfinder	H-scan	Sweepfinder	H-scan
African	0.052	0.076	0.032	0.054
Asian	0.303	0.613	0.155	0.494
European	0.582	0.701	0.626	0.252

Selection scan results for region around the FY*O mutation. Numbers indicate empirical p-value for Sweepfinder and H-scan statistics in 20kb and 100kb regions centered on the FY*O mutation. These are calculated by comparing the FY*O region statistics with the distribution of statistics from the whole genome. Table includes an average of results from each of the fifteen 1000 Genomes populations and results compare regions of similar recombination rate.

We find that FY*O has the largest population differentiation, as measured by F_*ST*_, of any SNP in the genome among the 1000 Genomes populations. This signature extends to the 100 kb region surrounding FY*O, though it is reduced to the 96.8th percentile. This supports our qualitative analysis of extreme population differentation. Both Sweepfinder and H-scan detect elevated scores indicative of selection in the 100 kb region, though DARC is not an outlier (Table 1, S4–S6 Tables). For example, using Sweepfinder, a method designed to detect recently completed hard selective sweeps based on the site frequency spectrum, the region is in the 97th percentile (corresponding to P-value = 0.032 in Table 1) genome-wide in African populations. Similarly, using H-scan, a statistic designed to detect hard and soft sweeps via pairwise homozygosity tract lengths, we find the DARC region in the 95th percentile in African populations (corresponding to P-value = 0.052 in Table 1). We note however that accumulation of diversity and elevated recombination rate (average rate 3.33 cM/MB in 5kb region) may reduce the power of these statistics. As African populations are almost exclusively FY*O, these selection results on African populations indicate significant and near significant signs of selection on FY*O. However, these results are much less extreme than the F_*ST*_ results indicating the presence of selective processes for which these tests may not have sufficient power, such as an ancient selection event.

We also compared extended haplotype homozygosity (EHH) [55] and integrated haplotype score (iHH) [29] in the region for each of the three allelic classes (S1 Fig), to investigate potential differences in linkage disequilibrium between FY alleles. EHH in the FY*B samples decreases rapidly with genetic distance, while the FY*A and FY*O samples show higher levels linkage disequilibrium. When examining EHH separately for each of the two major FY*O haplotype backgrounds, we find increased linkage disequilibrium, as expected. FY*O maintaining higher levels of linkage disequilibrium is in line with positive selection, particularly because African populations are known to have the lowest linkage disequilibrium among continental groups.

#### Evidence of positive selection at FY*A

Evidence for positive selection at the FY*A allele is currently under debate; binding assays show decreased binding of *P.vivax* to FY*A [18], though studies of the incidence of clinical malaria reach differing conclusions [12, 18, 19, 21]. Despite this debate, it exhibits strong population differentiation and structure. FY*A is present at high frequency in Europe, Asia and southern Africa, but is conspicuously absent from the rest of sub-Saharan Africa. Similar to FY*O, FY*A has a very high *F_ST_* (99.99th percentile); however, selection scans based on the site frequency spectrum and linkage disequilibrium fail to detect selection (Table 1, S6 and S7 Tables). In Asian samples, which are about 90% FY*A, H-scan is in the 51st percentile, while Sweepfinder is slightly elevated to the 85th percentile.

We further analyzed the frequency trajectory of FY*A over time utilizing ancient genomes. We find that FY*A maintains a 30–50% frequency in our samples throughout most time periods and geographic regions, indicating that FY*A was already common in Eurasia as early as the Upper Paleolithic (S3 Fig). We note these frequencies are substantially lower than those observed in contemporary East Asian populations. However, most of the Bronze Age Asian samples are from the Altai region in Central Asia, which have been shown to derive a large fraction of their ancestry from West Eurasia sources [56]. We also note that the only published ancient African (Ethiopian) genome is heterozygous for the FY*A allele, indicating FY*A was likely not introduced into East Africa due to recent back migration [50].

Taken together, we find that tests for positive selection on the FY*A allele are inconclusive. We observe a high F_*ST*_ and it is definitely ancient (likely present in human populations prior to the out-of-Africa expansion), but neither Sweepfinder nor H-scan gave significant results for signatures of selection. Given the less than clear association between FY*A and disease resistance, as well as the absence of strong signatures suggestive of natural selection shaping this locus we focus our detailed analysis to FY*O.

### Inference of T_MRCA_ of FY*O and FY*A

We were interested in estimating the age of the FY*A and FY*O alleles and the start time of selection for FY*O, based on the average number of pairwise differences between haplotypes. For FY*O, we initially estimate the time to most recent common ancestor (T_MRCA_) over all FY*O haplotypes to be 230,779 years (95% CI: 169,790–291,039 years), which would imply very old selection under the assumption of a *de novo* sweep model. We note however that both the presence of two deeply diverged haplotypes with low levels of within-group diversity, as well as observed recombination between FY*O and the FY*A/FY*B alleles may artificially increase the estimated T_MRCA_. We therefore devised a strategy to remove the effects of recombination and estimate the T_MRCA_ separately on each of the two major haplotypes, in order to obtain an approximate estimate for the start time of selection under the standing variation model. We estimate the major FY*O haplotype class to be 42,183 years old (95% CI: 34,100–49,030) and the minor haplotype class to be 56,052 years old (95% CI: 38,927–75,073) (S8–S10 Tables). For the FY*A allele, the allele age was estimated as 57,184 years old (95% CI: 47,785–64,732). Variation between population-specific T_MRCA_ estimates was low. Additionally, we find that Ust’-Ishim, a 45,000 years old individual from Siberia [51] is heterozygous for FY*A. Under the assumption of no recurrent mutations, this would set a minimum age of 45,000 years for the FY*A mutation. These results corroborate our hypothesis that FY*O was an ancient sweep, likely tens of thousands of years older than most other mutations associated with malaria resistance [57]. These T_MRCA_ estimates were used as a guide to seed our simulations for the following analysis; however, selection scenarios were not limited to these times.

### Mode and magnitude of positive selection on FY*O

FY*O’s two divergent haplotypes indicate it may have reached fixation in Africa via selection on standing variation. To investigate this, we utilized an Approximate Bayesian Computation (ABC) approach to estimate the magnitude of FY*O’s allele frequency at selection onset, followed by the selection coefficient (*s*) of FY*O.

To infer the magnitude of FY*O’s allele frequency at selection onset, we compared the posterior probability of five models of initial frequency at selection onset (*de novo* mutation (1/2N), 0.1%, 1%, 10%, 25%), utilizing a Bayesian model selection approach in ABC, based on Peter et al. (2012) [58–61]. It is important to remark that we use additional summary statistics in our ABC implementation, including commonly used scans of selection. We realized that the original method proposed by Peter et al. (2012) [58] did not include any of these statistics but, as we show in this work, those prove to be highly informative of the process. Briefly, for each model we ran 100,000 simulations based on the African demographic model inferred in [62] and centered on an allele with selection coefficient drawn from the distribution 10^*U*(−3,−0.5)^. We assumed an additive selective model, as empirical studies predict heterozygotes have intermediate protection against *P. vivax* infection [11, 12] and a selection start time similar to the estimated T_MRCA_ of FY*O’s major haplotype (40 kya). We investigate our power to distinguish between the different models utilizing cross validation. We show that we have high power to distinguish between *de novo* and higher initial frequencies, though there is some overlap between adjacent models (S1 Appendix). Utilizing a multinomial logistic regression method, we observed strong support for the 0.1% initial frequency model and low support all other models (posterior probabilities: *de novo* 0.0002; 0.1% 0.9167; 1% 0.0827; 10% 0.0000; 25% 0.0004)(S1 Appendix). We found these results to be robust to a range of recombination rates, selection start times and demographic models (Table 4 in S1 Appendix). We conclude selection on FY*O occurred on standing variation with a very low (0.1%) allele frequency at selection onset.

**Fig 3:**
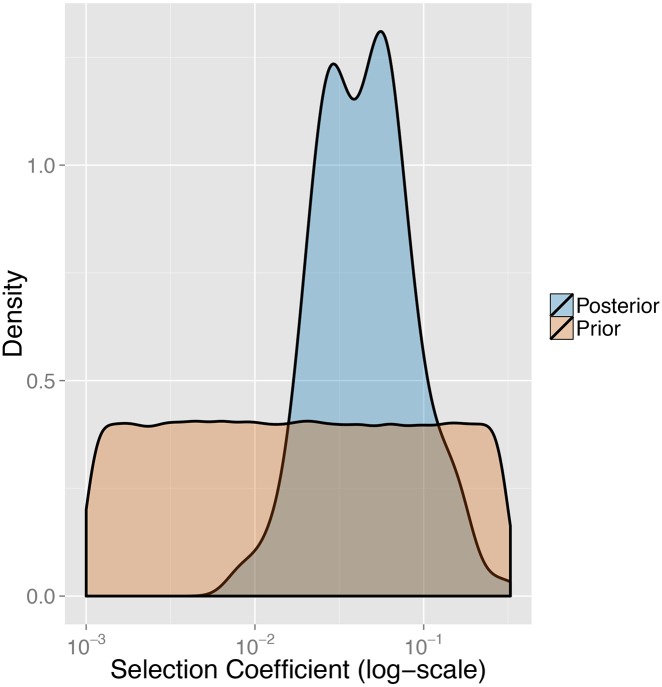
FY*O selection coefficient results. Prior and posterior distributions of FY*O selection coefficient.

We next sought to infer the strength of the selective pressure for FY*O. We estimated FY*O’s selection coefficient via ABC and local linear regression, assuming an allele frequency at selection onset of 0.1%. We find we have reasonable power to accurately infer *s* from these simulations; estimated and true selection coefficients have an *r*^2^ value of 0.85 with a slight bias of regression to the mean (Fig 3 in S1 Appendix). We estimate the selection coefficient to be 0.043 (95% CI: 0.011–0.18) (Fig 3).

To validate our model choice, we sampled selection coefficients from this posterior distribution and ran simulations with the initial frequency drawn from either 10^*U*(−5,−0.5)^ or *U*(0,1). With the log-based prior distribution, we re-estimate the initial frequency at 0.15% (95% CI: 0.018–0.77%; Fig 4 in S1 Appendix), closely fitting our inference. With the uniform prior distribution, we have much lower power to estimate initial allele frequency and we re-estimate the initial frequency at 6.86% (95% CI: -20.3–51.6%)(Fig 5 in S1 Appendix). This is not surprising as it has previously been shown that it is very difficult to estimate initial frequency with a uniform prior [58].

### Allelic classes of southern Africa

We also sought to understand the history of these alleles in southern Africa as, unlike equatorial Africa, malaria is not currently endemic in southwestern Africa and past climate was potentially unsuitable for malaria. Thus, we expect there was a lower or no selection pressure for FY*O or FY*A in this region. We analyzed sequences from the Bantu-speaking Zulu and indigenous ≠Khomani San. We find all three allelic classes are present in both populations (Zulu: FY*A 6%, FY*B 16%, FY*O 79%; ≠Khomani San: FY*A: 35%, FY*B 44%, FY*O 21%). The KhoeSan peoples are a highly diverse set of southern African populations that diverged from all other populations approximately 100 kya [63], and the ≠Khomani San represent one of the populations in this group. The Zulu population is a Bantu-speaking group from South Africa; southern Bantu-speakers derive 4–30% KhoeSan ancestry [64] from recent gene flow during the past 1,000 years. We first ask if the FY*O allele in the KhoeSan group represents recent gene flow from Bantu-speakers or whether FY*O has been segregating in southern Africa for thousands of years. We investigated global and local ancestry differences between FY*O carriers and non-carriers. We find a significant difference in genome-wide western African ancestry in ≠Khomani San FY*O carriers vs. non-carriers (17% average in FY*O carriers vs. 5.4% average in non-FY*O carriers, *p* = 0.014 based on a Wilcoxon Rank-Sum test). We also find a significant enrichment of local Bantu-derived ancestry around the FY*O mutation in the ≠Khomani San FY*O carriers (*p* = 2.78*10^−12^ based on Fisher’s exact test; S4 and S5 Figs). Each of these factors indicate that FY*O was recently derived from gene flow into the ≠Khomani San population from either Bantu-speaking or eastern African groups. We then explored the relationship of FY*O in KhoeSan and Zulu samples to Bantu-speaking populations from equatorial Africa. A haplotype network of the ≠Khomani San FY*O carriers indicated that each 20kb haplotype was identical to a haplotype from populations further north (S6 Fig). We tested the Zulu FY*O samples as well, and found that they have identical, though more diverse, haplotypes than other Bantu-speaking populations (Fig 2). However, the increase in diversity may be due to calling biases and recombination between different allelic classes in the Zulus (see discussion).

We then sought to understand the prehistory of FY*A in southern Africa. The FY*A allele is common in San populations, despite its absence from equatorial Africa (Fig 1). We compared the FY*A haplotypes found in the ≠Khomani San and Zulu populations with FY*A haplotypes present in Asia and Europe to distinguish between three hypotheses. The FY*A mutation in southern Africa either was 1) segregating in the ancestral human population, 2) due to recent admixture from migrations ‘back to Africa’, or 3) arose convergently in a separate mutation event distinct from the European / Asian mutation. We find that Zulu FY*A haplotypes are highly diverse; some are identical to non-African FY*A haplotypes, while others are unique or ancestral (Fig 2). Global ancestry results show no statistically significant difference between Bantu or KhoeSan ancestry in FY*A ≠Khomani San carriers and non-carriers based on a Wilcoxon Rank-Sum test (San: *p* = 0.85, Bantu: *p* = 0.101). Our local ancestry results indicate that FY*A carriers are significantly enriched for San ancestry around FY*A compared with non-carriers based on Fisher’s exact test (*p* = 0.011). Our results support hypothesis (1), i.e. high ≠Khomani San FY*A haplotype diversity indicates FY*A has an ancient presence in southern Africa. Furthermore, as Bantu-speaking populations from equatorial Africa currently are exclusively FY*O, it is unlikely they transferred FY*A to KhoeSan after the Bantu expansion. Rather, the FY*A haplotypes in the Zulu are largely derived from admixture with the indigenous KhoeSan populations, or potentially very recent gene flow from European/Asian immigrants to South Africa.

## Discussion

The FY*O allele in DARC is often cited as a quintessential example of positive selection in the human genome due to its disease resistance association and extreme continental *F_ST_*. However, the population genetics and evolutionary history of DARC remains understudied. Here, we infer that the FY*O mutation in Africa underwent a ancient, soft selective sweep in equatorial Africa from multiple lines of evidence:

- Two divergent haplotypes forming separate star-like phylogenies
- Both divergent FY*O haplotypes are found in hunter-gatherer and Bantu populations
- Ancient T_MRCA_ estimates of FY*O haplotypes
- Low frequency of FY*O in southern Africa samples introduced by recent gene flow
- Extreme population differentiation, but reduced signatures of selection in surrounding region
- ABC estimates of FY*O consistent with a low initial frequency and a high selection coefficient

In what follows, we explain how these different lines of evidence describe a complex picture of evolution at this locus. First, we identify two divergent haplotypes carrying the FY*O mutation, an observation that is consistent with previous results [27, 28]. These haplotypes, defined by four SNPs (one 600 bps upstream and three within 2500 bps downstream), are not compatible with a simple hard sweep model where one haplotype sweeps to fixation due to a positively selected *de novo* mutation. These two haplotypes both form star-like phylogenies and do not exhibit geographic structure in equatorial Africa, indicating that both haplotypes were selected for in the same regions.

Second, identification of identical haplotypes in highly divergent African populations implies an ancient selective sweep before the complete divergence of these populations. The first line of evidence for this scenario is that Baka and Mbuti populations have identical FY*O haplotypes in similar proportions as the Bantu populations. This is relevant because Baka and Mbuti are hunter-gatherer populations that diverged a long time ago from Bantu African populations (50–65 kya) as well as from each other (20–30 kya) [47, 65–68]. Secondly, we observe low levels of admixture between these groups (Bantu admixture in Mbuti: 0–16%, Bantu admixture in Baka: 6.5–9.4%) [69, 70]. However as many individuals were estimated to have no Bantu admixture and FY*O is nearly fixed in these hunter-gatherer populations, these identical haplotypes are unlikely to be due to recent gene flow. All together these observations are consistent with the mutation sweeping before or during the hunter-gatherer / Bantu split. This observation, along with the ancient T_MRCA_, is consistent with selection acting on this locus from ancient times. We note that low levels of gene flow may have resulted in its fixation due to its selection coefficient; however, we found no increase of Bantu local ancestry around FY*O in the Baka hunter-gatherers, indicating that this may not be the case (S7 Fig).

Third, the confidence intervals of our FY*O T_MRCA_ estimate of FY*O’s major haplotype overlap the divergence times estimated for the hunter-gatherer / Bantu split, supporting the idea that the sweep occurred just before or during the split, potentially as the common ancestral population first dispersed into equatorial forest.

Fourth, FY*O’s much lower frequency in the ≠Khomani San, as well as other KhoeSan populations [9], indicates that it may have had a lower selective pressure in southern Africa. The past and current arid climate have made southern Africa a poor habitat for mosquitoes, reducing the associated risk of infection [71]. Furthermore, local and global ancestry results indicate that FY*O may be due to recent gene flow into these populations, as ≠Khomani San FY*O carriers are significantly enriched for global Bantu ancestry and local Bantu ancestry in the FY*O region, relative to non-carriers.

Fifth, the high *F_ST_*, coupled with lower Sweepfinder and H-scan statistics, indicate an ancient sweep and/or selection on standing variation. A recent hard sweep in Africa would drastically reduce variation around the selected site (resulting in high homozygosity estimated from H-scan) and shift the site frequency spectrum to high and low frequency sites (inferred as selection by Sweepfinder). Instead, slightly lower H-scan and Sweepfinder statistics indicate more diversity and less extreme site frequency spectrum shifts than expected in a recent hard sweep. This may be due to an ancient sweep that had time to accumulate diversity and/or a sweep on standing variation that increased the frequencies of multiple diverse haplotypes. Selection on standing variation has been shown to have wider variance in relevant summary statistics and methods for detecting selection. The variance size depends on parameters such as allele frequency, time of selection, and strength of selection [72].

Sixth, ABC estimates initial FY*O frequency of magnitude 0.1% and selection coefficient 0.043 (95% CI:0.011–0.18). Though this initial frequency magnitude is very low, it drastically increases the probability that an allele of this selection coefficient will fix in the population, relative to a *de novo* mutation (see below).

### FY*O and FY*A T_MRCA_ estimates

We estimate the T_MRCA_ of all FY*O haplotypes to be 230,779 years (95% CI: 169,790–291,039 years) and the T_MRCA_ of the most common haplotype class to be 42,183 years (95% CI: 34,100–49,030 years). We note that two of the assumptions of our estimation method (no recombination and star-like phylogeny) are partially violated in our data. However, we developed a strategy to mitigate the effect of recombination (see Methods) and only the T_MRCA_ estimation with all FY*O haplotypes differs greatly from a star-like phylogeny (due to the deep divergence in the two main haplotypes). Estimates of T_MRCA_ are also prone to large confidence intervals due to the stochasticity of the allele frequency trajectory, but all estimates indicate the FY*O mutation is older than most known malaria resistance alleles [57]. Previous estimates of the time of fixation of the FY*O mutation, based on lower density data, range from 9–63 kya (adjusted to our mutation rate and generation times) [28, 73]. Other T_MRCA_ estimates ranging from 9 to 14 kya were calculated on microsatellites linked to FY*O [73], which seem to have underestimated the age of the mutation. Perhaps the most comprehensive work on this problem until now was by Hamblin and DiRienzo [28], who estimated the time to fixation of FY*O to be 63 kya (95% CI: 13,745–205,541 years; converted to our mutation rate). This is older than our estimates, but has overlapping confidence intervals. More recently, Hodgson et al. [74] estimated the time necessary for FY*O’s frequency to increase from 0.01–0.99 to be 41,150 years, based on an inferred selection coefficient in Madagascar.

We inferred FY*A to be an ancient mutation, likely segregating throughout Africa before FY*O swept to fixation. We estimate FY*A to be 57,187 years old (95% CI: 47,785–64,732 years), 15,000 years older than the most common FY*O haplotype and overlapping estimates of the out-of-Africa expansion time [62, 75–77]. We note that the San FY*A haplotypes were not used in this T_MRCA_ calculation as there were few homozygous sequenced FY*A San samples and we confined our estimates to homozygotes to reduce issues due to phasing errors. As we are only looking at the out-of-Africa diversity of FY*A, it is likely this T_MRCA_ is more indicative of FY*A’s expansion during and after the out-of-Africa event. Ancient DNA from a Paleolithic hunter-gatherer provides evidence that FY*A was already present in Eurasia by at least 45,000 years ago, thereby setting a lower bound for the age of the mutation. Its intermediate frequency in ≠Khomani San and Zulu populations, and similar haplotypic structure is consistent with FY*A existence in Africa at an appreciable frequency before the out-of-Africa expansion had occurred. The deep divergence of the ≠Khomani San from all other tested populations carrying FY*A strongly supports this ancient origin.

### Scaling parameter uncertainty

Our results are scaled with the mutation rate of 1.2 * 10^−8^ mutations / basepair / generation and a 25 year generation time. This mutation rate is supported by many previous whole-genome studies ([51, 78–81]; range: 1 − 1.2 * 10^−8^ mutations / basepair / generation), but we are aware of recent studies suggesting a higher mutation rate that are either based on exome data ([82–84]; range: 1.3 − 2.2 * 10^−8^ mutations / basepair / generation) or whole-genome data ([85, 86]; range: 1.61 − 1.66 * 10^−8^ mutations / basepair / generation). To take into account this uncertainty, we performed additional analyses using a mutation rate of 1.6 * 10^−8^ mutations / basepair / generation. With this higher rate, we estimate more recent coalescent times of the FY*O and FY*A mutations; specifically we would estimate the FY*O T_MRCA_ to be 32 kya (vs. 42 kya) and the FY*A T_MRCA_ to be about 43 kya (vs. 57 kya). It is important to consider that most quantities in population genetics are scaled by the mutation rate and effective population size. Therefore, any changes in the mutation rate result in changes not only in our T_MRCA_ estimates, but also in the timescale of the split between African and non-African populations. For example, a recent study of the divergence between African and non-Africans, estimates a median of divergence between 52–69 kya and a final split around 43 kya, using a mutation rate of 1.2 * 10^−8^ mutations / basepair / generation [76]. If we use a higher mutation rate of 1.6 * 10^−8^ mutations / basepair / generation the median divergence would be 39–52 kya with a final split around 33 kya. Thus, regardless of the mutation rate (and the corresponding demographic scenario), we estimate the FY*O mutation to have occurred soon after the estimated final split.

### FY*O initial frequency and selection coefficient estimations

FY*O’s two divergent, common haplotypes in Africa indicate it may have reached fixation due to selection on standing variation. We infer that the FY*O mutation underwent a selective sweep on standing variation with a selection coefficient comparable to some of the most strongly selected loci in the human genome [57]. Utilizing a Bayesian model selection approach implemented in an ABC framework, we find that FY*O likely rose to fixation via selection on standing variation; though the frequency of FY*O at selection onset was very low (0.1%). We estimate FY*O’s selection coefficient to be 0.043 (95% CI: 0.011–0.18), consistent with previous estimates (>0.002 in the Hausa [28], 0.066 in Madagascar [74]). The similarity of these results indicates FY*O may have a similar selective effect in diverse environments.

This selection coefficient is similar to other loci inferred to have undergone strong selection in the human genome, including other malaria resistance alleles [57]. The selection coefficients of these other malaria resistance alleles were inferred via a variety of different methods, mostly utilizing simulations and a rejection framework. Our understanding of human demographic history has improved over the past few years with the increase of genomic data. Previous estimates did not consider realistic demographic models, while we utilized the African demographic model inferred in Gravel et al. [62]. Assuming the standard neutral model when the true demography is more complex may result in overestimating the selection coefficient for some of the regions mentioned in Hedrick et al. 2011 [57] (due to recent population expansions).

At first glance it would be reasonable to consider such a low initial frequency equivalent to a scenario of selection on a *de novo* mutation. In order to distinguish between the two possibilities we use the diffusion approximation by Kimura [87, 88] to estimate the probability of fixation (equation 8 in [88]) and demonstrate that it is much more likely to reach fixation with an initial frequency of 0.1% than a scenario of a new mutation arising in the population. We find that an allele with selection coefficient 0.043 and initial frequency 0.001 has a 99.4% probability of fixing, while a *de novo* mutation with the same *s* has only an 8.2% probability of fixing. It is important to note that in our calculation the initial frequency (*p*) in the equation for the *de novo* mutation scenario is calculated using the effective population size, as opposed to the census population size. However, if we reasonably assume *N* ≥ *N*_*e*_, *p* is likely at least 0.1% in the population. This translates in our estimates for the probability of fixation of a *de novo* mutation being far more optimistic than expected if the ancestral African census population size was much larger than the effective size. This low initial frequency until 40 kya is consistent with FY*O’s absence from non-African present and ancient genomes.

It is important to note that selection on standing variation and a soft sweep are not necessarily synonymous. Selection on standing variation (the model we are testing) asks about the frequency of the allele at selection onset. However, it is agnostic to the number of haplotypes that are actually picked up at selection onset. A soft sweep states that multiple haplotypes are picked up at selection onset. Via our *msms* simulations, we are unable to say, for each individual simulation, whether just one haplotype was picked up or if multiple haplotypes were picked up (either due to additional mutations or recombination). However, we note that of our accepted simulations, summary statistics of the 0.1% model are much more diverse and much more similar to our data, than the *de novo* model. We speculate that this may be due to multiple haplotypes that are being picked up in the 0.1% model.

### FY*O and FY*A mutations and *P. vivax*

FY*O and FY*A are thought to be under positive selection due to *P. vivax*, a malaria-causing protozoan that infects red blood cells through the Duffy receptor. Individuals with the FY*O allele do not express the Duffy receptor in red blood cells resulting in immunity to *P.vivax* [6, 10] and individuals with the FY*A allele may have lower infectivity rates [11, 12, 18–21]. Unlike *P. falciparum*, the most common and deadly malaria protozoan in Africa that uses multiple entry receptors, *P.vivax*’s one mode of entry allows the possibility of resistance with only one SNP.

Was *P.vivax* the selective pressure for either the FY*O or FY*A mutations? *P. vivax* is currently prevalent in equatorial regions outside of Africa; however it is unknown if *P. vivax* has ever been endemic to Africa. There is an ongoing debate as to if *P.vivax* originated in Asia or Africa. Previously, it was thought *P.vivax* originated in Asia, as Asian and Melanesian *P.vivax* has the highest genetic diversity [42, 89] and the most closely related parasite to *P. vivax* is *P. cynomolgi*, a macaque parasite [40, 42]. However, recent evidence shows global human-specific *P.vivax* forms a monophyletic cluster from *P. vivax*-like parasites infecting African great apes, suggesting an African origin [40].

Human-specific *P.vivax* sequences form a star-like phylogeny likely due to a relatively recent demographic expansion. Our T_MRCA_ estimates of human-specific *P.vivax* sequences are 70–250 kya (S12 Table), consistent with previous estimates (50–500 kya, [42, 43, 89]). As the T_MRCA_ of human-specific *P.vivax* is estimated to be before or overlapping the T_MRCA_ of FY*O, this is consistent with the hypothesis of *P.vivax* being the selective agent responsible for the rise of FY*O in Africa. However, there are two possible scenarios that could explain the T_MRCA_ estimates for *P. vivax*. A first scenario is that the estimated T_MRCA_ of human *P.vivax* indicates the start of the association between host and parasite, thus marking the start of selective pressure on the host. A second scenario is that these estimates overlap the human out-of-Africa expansion times. It is possible that human-specific *P.vivax* expanded out of Africa with humans, resulting in the estimated T_MRCA_ for *P. vivax*. The human *P.vivax* currently in Africa could be from recent migration [43, 89].

Additionally, it is yet unclear if such a high selection coefficient is consistent with the fact that the general severity of *P. vivax* is currently much lower than that observed for *P. falciparum*, causing more morbidity than mortality. The combination of these observations lead us to suggest that further work is necessary to better understand the evolutionary history of *P. vivax* to reconcile the demographic scenarios that could have given rise to such a complex pattern.

All together, our results suggest that the evolutionary history of the FY*O mutation, a single SNP under strong selection in human populations, has been a complex one. Multiple haplotypes present in highly divergent African populations are consistent with selection on standing variation, shaping the evolution of this locus that was present in very low frequency in ancestral populations. Although more work needs to be done to understand how *P.vivax* may have shaped the evolution of this locus, our results provide a framework to evaluate the evolution of the parasite and formulate specific hypotheses for its evolutionary history in association with its human host.

## Materials and methods

### Genetic data and processing

#### Modern population sequence data

Data used in this study was retrieved from the African Genome Variation Project (AGVP, Zulu, Bagandans), Human Genome Diversity Project (HGDP, Mbuti [90, 91]), the 1000 Genomes Project [92], as well as data sequenced here (Sikora et al. In Prep, Nzebi, Baka) and ≠Khomani San. Sequence data for 1000 genomes populations was retrieved from the phase 3 version 3 integrated phased call set (ftp://ftp-trace.ncbi.nih.gov/1000genomes/ftp/release/20130502/). Related individuals were removed (ftp://ftp-trace.ncbi.nih.gov/1000g/ftp/release/20130502/20140625_related_individuals.txt).

SNPs from samples sequenced in-house, the AGVP and the HGDP were recalled together. Bam files from the AGVP were downloaded from the EGA archive via a data access agreement. Chromosome 1 bam files for all three data sources were cleaned with SamTools [93]. The following protocols were run to prepare the bam files: CleanSam.jar, FixMateInformation.jar, ValidateSamFile.jar, SortSam.jar, and MarkDuplicates.jar. We applied GATK [94] base quality score recalibration, indel realignment, and duplicate removal. We performed SNP discovery with GATK UnifiedGenotyper (default settings and min_conf = 10) and variant quality score recalibration according to GATK Best Practices and a tranche sensitivity threshold of 99% [95, 96]. SNPs were phased and imputed by Beagle in two steps [97]. First, the 1000 Genomes sequences were used as a reference panel to phase and impute SNPs present in both datasets. Next, Beagle was run a second time without a reference panel to phase and impute remaining SNPs. The 20 kb region surrounding FY*O (chr1:159,164,683-159,184,683) was extracted from the 1000 Genomes data and merged with the recalled data. We identified 401 SNPs in the merged dataset. Analyses were restricted to biallelic SNPs. Derived and ancestral allelic states were determined via the human ancestor sequence provided by ensembl from the 6 primate EPO [98]. SNPs without a human ancestor were not included in analyses.

#### Ancient genomes

Ancient genomes were processed as described in Allentoft et al. [56]. Briefly, we randomly sampled a high quality read for each ancient individual with coverage at the Duffy SNPs. Population allele frequencies were then estimated by combining multiple individuals into populations as in Allentoft et al. [56].

#### Great ape sequence data

Great ape sequences mapped to their species-specific genomes from Prado-Martinez et al. [99] were utilized in this analysis. This included 24 chimpanzees (panTro-2.1.4), 13 bonobos (panTro-2.1.4), 24 gorillas (gorGor3), and 10 orangutans (ponAbe2). The DARC gene and 1kb surrounding region was extracted from each species based on Ensembl annotations: gorilla (chr1:138,515,328-138,517,811), and chimpanzees and bonobos (chr1:137,535,874-137,538,357) [100]. Orangutans were excluded from analyses because they have two regions orthologous to the human DARC gene (chr1:92,205,245-92,206,855, chr1_random:12,168,081-12,170,200,). SNP functionality was annotated by SNPEff [101].

### Population structure analyses

#### Haplotype analyses

Median-joining networks were constructed via popArt [102].

#### Promoter region summary statistics

Summary statistics (number of segregating sites, average number of pairwise difference, Tajima’s D) were calculated in the 750 bp promoter region upstream every genes in the 1000 Genomes integrated data via VCFtools [103]. The summary statistics from DARC’s promoter region were compare to all other promoter regions. Gene locations were extracted from ensembl release 72 [100].

#### Selection summary statistics

We analyzed methods in three main categories of selection detection: population differentiation (F_*ST*_), site frequency spectra (Sweepfinder [52, 53]), and linkage disequilibrium (H-scan [54]). Genomic regions that have undergone a recent hard selective sweep are expected to have site frequency spectrums skewed toward rare and high frequency derived variants, increased homozygosity and, if local adaptation, high population differentiation. Summary statistics were calculated for the fifteen 1000 Genomes populations.

Weir and Cockerham’s (1984) weighted *F_ST_* was calculated in VCFtools [103, 104]. Sweepfinder, a method designed to detect recent hard selective sweeps based on the site frequency spectrum was ran via the SweeD software [52, 53]. H-scan, a statistic designed to detect hard and soft sweeps [54], measures the average length of pairwise homozygosity tracts in a population. By utilizing pairwise homozygosity tracts, this method can detect soft sweeps, sweeps that have resulted in multiple haplotypes reaching high frequency. The default distance method was used (-d 0) and the maximum gap length between SNPs was set to 20kb. To calculate recombination adjusted results, recombination rates from deCODE [105] were lifted over from hg18 to hg19. We limited comparisons to regions with average recombination rates within 25% of the DARC region’s recombination rate. EHH was calculated via the R package rehh [106].

#### Inference of T_MRCA_

We estimated the T_MRCA_ of the FY*A and FY*O mutations through a method based on the average number of pairwise differences between two haplotypes [107]. We used the equation, $\hat{T}=\frac{\pi }{\mu }$, where *π* is the average number of pairwise differences per base pair in the sample and *μ* is the mutation rate per year per base pair. We assumed a mutation rate of 1.2 * 10^−8^ mutations per basepair per generation and a generation time of 25 years. Analyses were restricted to individuals homozygous for FY*B, FY*A or FY*O due to phase uncertainty. Regions were limited to “callable” sequence based on the 1000 genomes strict mask. 18,333 basepairs were callable in the 20kb region. Standard error estimates were calculated by 1000 bootstrap estimates with replacement.

This T_MRCA_ method calculates the average time to most recent common ancestor between two haplotypes in the sample. It assumes a star-like phylogeny and no recombination. Our phylogenies are close to star-like (S2 Fig) and Slatkin and Hudson [107] show that near star-like phylogenies, with *N*_0_ * *s* >> 0, result in valid allele age estimates (where *N*_0_ is the effective population size and *s* is the selection coefficient). We estimate the T_MRCA_ of FY*O’s two major haplotypes separately as their deep divergence would strongly violate the star-like phylogeny requirement. We focused on a star-like phylogenetic method, as opposed to the coalescent, as the latter does not take into account selection, an apparently strongly influencing effect in this region, and thus would result in an artificially much older T_MRCA_ estimate.

We developed a variation of this method to account for recombination exhibited between allelic classes. For each pair of haplotypes, we identified the maximum region around the focal SNP with no signs of recombination between these haplotypes and haplotypes of other allelic classes. To identify this region, we expanded out from the focal SNP until we identified a SNP that was segregating both in the haplotype pair and in any samples in other allelic classes. This SNP is identified as a potential recombinant. The region for comparison is then set to the region between the two farthest nonrecombinant SNPs on each side plus half the region between the last nonrecombinant SNP and the first potential recombinant SNP. To calculate pairwise T_MRCA_, we count the number of nucleotide differences between the two haplotypes in this region. All pairwise T_MRCA_ estimates are then averaged to estimate sample T_MRCA_.

Minimum and maximum region sizes were also set. The minimum total sequence length was set to 3,000 basepairs, to ensure the expected number of mutations is at least one. This is important because if, for example, the SNPs adjacent to the focal SNP are both potential recombinants, the estimate allele age from these haplotypes would be 0, biasing the estimate to a more recent time. A maximum region size is set because the signature of selection decays as distance increases from the focal SNP, likely due to unseen recombination events. The maximum distance on each side was set to the distance in which EHH fell below 0.5 or 0.66 (FY*O 0.5: 3,322 bps upstream, 3,034 bps downstream; FY*O 0.66: 2,640 bps upstream, 3,034 bps downstream; FY*A 0.5 and 0.66: 4,358 bps upstream, 1,176 bps downstream). In most cases, small variations in the size of the selected region have little effect on the results; however, it did result in two very different estimates for the FY*O minor haplotype due to a common SNP included in the larger region size. The estimates with the EHH 0.66 cutoff is 56,052 years (95% CI: 38,927 − 75,073), while with the EHH 0.5 cutoff is 141,692 years (95% CI: 117,979 − 164,918).

#### FY*O initial frequency and strength of selection

To estimate FY*O’s selection coefficient and initial frequency at selection onset in equatorial Africa (based on the LWK population), we utilized an Approximate Bayesian Computation (ABC) approach in two steps: (1) we identified the best model of FY*O frequency at selection onset (based on [58]) and (2) we estimated the selection coefficient assuming that initial frequency.

Inference was based on simulations, via *msms* [108], of 5 kb sequences centered on a selected allele with the African demographic model inferred in Gravel et al. [62]. Adapted to the mutation rate (1.2 * 10^−8^ mutations / basepair / generation) used it this paper, this model has an ancestral effective population size of 14,376 individuals that increases to 28,470 individuals 291,000 years ago. We allow the allele to evolve neutrally until 40 kya (rounded T_MRCA_ of major FY*O haplotype class), at which point we assumed a constant additive model of selection (using the -SI *msms* flag). For all simulations the prior distribution for the selection coefficient was *s* = 10^*U*(−3,−0.5)^. The recombination rate was inferred from the average for the 5kb region from the deCODE map (3.33 cM/MB) [105]. We assumed a mutation rate equal to 1.2 * 10^−8^ mutations per base pair per generation and a generation time of 25 years.

First, we utilized a Bayesian model selection approach in an ABC framework to estimate the magnitude of FY*O’s initial frequency at selection onset (implemented in the R package *abc* [59, 109, 110]). We compared five models of the initial FY*O frequency (*de novo* (1/2N), 0.1%, 1%, 10%, 25%). We ran 100,000 simulations for each model and recorded summary statistics: *π* (average number of pairwise differences), number of segregating sites, Tajima’s D, Fay and Wu’s *θ*_*H*_, number of unique haplotypes, linkage disequilibrium (average EHH centered on the selected site at the two ends of the sequences and iHH), allele frequency statistics (number of fixed sites, singletons, doubletons, singletons / fixed sites), H statistics [111] (H1, H2, H12, H2/H1), and final frequency of the selected allele. Summary statistics were centered, scaled, and transformed with PLS-DA to maximize differences between models, and we retained the top 5 PLS-DA components (mixOmics R package [112], similar to [58]). We then utilized a multinomial logistic regression method with a 1% acceptance rate to estimate the posterior probability of each model.

Second, based on the model with the highest posterior probability (initial frequency: 0.1%), we estimated the selection coefficient using ABC and local linear regression. We ran 200,000 simulations and utilized the most informative summary statistics, as determined via information gain: number of segregating sites, number of mutations with more than two copies, number of fixed sites, (number of singletons) / (number of fixed sites), H1, H2, H12, number unique haplotypes, average EHH at ends, and the final frequency of the selected allele. We centered, scaled, and transformed these statistics with PCA, retaining PCs that explained 95% of the variance. Last, we estimated the posterior distribution with a logistic regression model and a 1% acceptance rate.

#### Allelic classes in southern Africa

This analysis utilized data from the Zulu [64] (Omni 2.5 array and low coverage sequence data, re-called with the rest of the African samples) and ≠Khomani San (550K and Omni Express and Omni Express Plus) and exome data). Exome data along with SNP array data (550k, Omni Express and Omni Express Plus) were merged with the HGDP set for the network analysis. We examined 84 KhoeSan and 54 Human Genome Diversity Panel (HGDP) individuals from 7 different populations [90]. There were 8 Pathan, 8 Mbuti Pygmy, 8 Cambodian, 8 Mozabite, 8 Yakut, 8 Mayan and 6 San individuals in the HGDP data set. The HGDP genotype data used in this study was acquired from Dataset 2 Stanford University and contained about 660,918 tag SNPs from Illumina HuHap 650K [113]). Exome data of the HGDP data set was previously sequenced and used in our analysis. Single nucleotide polymorphism (SNP) array/genotype and exome data were merged using PLINK. The SNP array platforms were merged as follows: HGDP650K, KhoeSan 550K OmniExpress and OmniExpressExomePlus. All individuals in the data set had full exome data and SNPs with a missing genotype rate more than 36% were filtered out of the data set.

Global San ancestry percentages were calculated from array data via ADMIXTURE [114]. For the ≠Khomani San samples, Europeans and a panel of 10 African populations from each major geographic region were used as potential unsupervised source populations. As the array data did not include rs2814778 or rs12075, these alleles were acquired from the corresponding exome data for each individual. Zulu global ancestry percentages are from [64] and FY status was determined from the corresponding sequence data. Only samples with matching identification numbers for the array and sequence data were included.

Local ancestry was determined using RFMix v1.5.4 [115]. For the ≠Khomani San samples, input files were array specific phase files, Omni Express and 550k, with three potential ancestral populations: (LWK) Bantu-speaking Luhya from Kenya, (CEU) western European, and (SAN) Namibian San. For the Zulu samples, we first merged and phased Omni2.5 genotype data for the two reference populations (Luhya (LWK) from Kenya and Nama (Khoe) from southern Africa) and the admixed population (Zulu). The Luhya data was downloaded from 1000 Genomes Project phase 3 (100 individuals) and the Nama genotype data is in preparation (102 individuals). The Zulu Omni2.5 file was downloaded from the African Genome Diversity Project and contained 100 individuals. Files were merged with PLINK and sites with missing genotype rate greater than 10% were filtered out. SHAPEIT v2.r790 was used to phase this merged data set [116]. For further phasing accuracy, family information was included for the Nama individuals and the—duohmm option was used when running the phase command; there were 7 duos and 1 trio included in our data set. After phasing, related Nama individuals were removed and only the Nama individuals with limited admixture were kept as the San reference for input into RFMix. When running RFMix, the PopPhased option was selected in the command; this option re-phases the original data, correcting haplotype phasing. Additionally, the command was run with two iterations. Local ancestry around the coding region of Duffy was extracted and plotted. A similar procedure was used to call local ancestry for the ≠Khomani San population using RFMix v1.5.4 [91].

We also constructed a median joining network (using Network [117]), for the 20kb region centered on the FY*O mutation. Site-specific weights were determined based on GERP conservation score. GERP scores were obtained from the UCSC genome browser (http://hgdownload.cse.ucsc.edu/gbdb/hg19/bbi/All_hg19_RS.bw) based on an alignment of 35 mammals to human. The human hg19 sequence reference allele was not included in the calculation of GERP RS scores. SNPs with an extremely negative GERP score (-5 or lower) were down-weighted to 5, SNPs with a GERP score higher than 3 were up-weighted to 15, and SNPs with a GERP score in-between these values were weighted to 10. The FY*O mutation was given a weight of 10, though it had a GERP score of 4.27. Maximum parsimony was used post calculation to clean the network by switching off unnecessary median vectors. The resulting network was drawn and edited in DNA publisher [117].

#### T_MRCA_ of *P.vivax* genes

We estimated the T_MRCA_ of human-specific *P. vivax* gene sequences from Liu et al. [40]. We assumed a star-like phylogeny and used the same pairwise differences equation as in the FY*O/FY*A estimates to calculate the T_MRCA_ of each *P.vivax* gene. We assumed a mutation rate of 5.07*10^−9^ basepairs per generation and a generation time of 1 year [89].

## Supporting information

S1 Appendix Supporting methods. Elaboration on the initial frequency & selection coefficient estimator.
(PDF)

S1 Fig EHH plots by allelic type. EHH plots for the 20kb region surrounding the FY*O mutation. A) FY*O samples centered on FY*O mutation B) FY*A samples centered on FY*A mutation C) FY*B samples centered on FY*O mutation D) FY*B samples centered on FY*A mutation.
(PDF)

S2 Fig Genetree genealogies. Geneology from Genetree of the 5kb region around FY*O. Dots indicate mutations and bottom numbers indicate number of samples with that haplotype. Left: geneology of FY*A samples from CHB population. Right: geneology of FY*O samples from LWK population.
(PNG)

S3 Fig Allele frequencies over time and space. Paleo: paleolithic; Hunter: hunter-gatherer; neol: neolithic; baEur: Bronze Age Europe; baStep: Bronze Age Steppe region; baAsia: Bronze Age Asia; ir: Iron Age. Sequences from Allentoft et al. (2015) [56].
(PDF)

S4 Fig Local ancestry around FY*O mutation in ≠Khomani San samples. A) Homozygous FY*B samples B) Homozygous FY*O samples C) Homozygous FY*A samples D) FY*O/FY*B samples E) FY*A/FY*B samples F) FY*A/FY*O samples.
(PDF)

S5 Fig Local ancestry around FY*O mutation in Zulu samples. There were no homozygous FY*A samples. A) Homozygous FY*B samples B) Homozygous FY*O samples C) FY*B/FY*O samples D) FY*A/FY*O samples.
(PDF)

S6 Fig Network image of 10 kb on either side of the Duffy locus. Weights are based on GERP conservation score. Asterisk indicates the root of the network. Blue circles indicate FY*O haplotypes.
(PNG)

S7 Fig Average fraction of Baka (red) and Nzebi (blue) ancestry along chromosome 1. Dashed line indicates position of the DARC gene. Local ancestry in Baka Pygmies was inferred using RFMix [115]). Due to the unavailability of an unadmixed source population panel for the Pygmy ancestry, we initially ran RFMix on a single Baka individual as target with the remaining individuals as source population. Local ancestry was then updated in both the Baka and Nzebi sources using four EM iterations.
(PDF)

S1 Table Samples included in study. (PDF)

S2 Table Allele frequencies by population. (PDF)

S3 Table Nucleotide diversity statistics. Nucleotide diversity statistics in the 5kb, 10kb, and 20kb region surrounding the FY*O mutation.
(PDF)

S4 Table Promoter region summary statistics. Summary statistics were calculated in 750 bp region upstream from DARC and compared to the 750 bp region upstream from all other genes in genome in each population. Summary statistics calculated: number of segregating sites (s), number of pairwise differences (*π*), and Tajima’s D. We quantified the percentile in the genome (Per.), median, and 95% confidence interval (CI).
(PDF)

S5 Table F_*ST*_ statistics. Weir and Cockerham’s weighted F_*ST*_ was calculated for each SNP in the genome and for 5 kb, 10 kb, and 20 kb windows. F_*ST*_ result and its percentile in the genome is reported for all fifteen 1000 Genomes populations.
(PDF)

S6 Table Sweepfinder statistics. We report the likelihood that the Duffy region (20 kb and 100 kb) underwent a recent hard selective sweep [52, 53]. This likelihood is determined via a composite likelihood ratio test where the numerator is the likelihood of the region site frequency spectrum given a hard selective sweep and the denominator is the likelihood of the region given a neutral model. This likelihood is compared to likelihoods from all other regions in the genome, as well as regions with average recombination rates within 25% of the Duffy region’s recombination rate.
(PDF)

S7 Table H-scan statistics. We report the maximum H-scan score for the Duffy region (20 kb and 100 kb). This score is then compared to the max score from all other regions in the genome, as well as regions with average recombination rates within 25% of the Duffy region’s recombination rate.
(PDF)

S8 Table T_MRCA_ results for FY*O major haplotype. Results for the T_MRCA_ of FY*O major haplotype by population. Results assume 25 year generation time and mutation rate of 1.2 * 10^−8^ mutations per basepair per generation. Confidence intervals are calculated from 1000 bootstrapped samples.
(PDF)

S9 Table T_MRCA_ results for FY*O minor haplotype. Results for the T_MRCA_ of FY*O minor haplotype by population. Results assume 25 year generation time and mutation rate of 1.2 * 10^−8^ mutations per basepair per generation. Confidence intervals are calculated from 1000 bootstrapped samples.
(PDF)

S10 Table T_MRCA_ results for FY*A haplotype. Results for the T_MRCA_ of FY*A by population. Results assume 25 year generation time and mutation rate of 1.2 * 10^−8^ mutations per basepair per generation. Confidence intervals are calculated from 1000 bootstrapped samples.
(PDF)

S11 Table Great ape DARC nonsynonymous mutations. All nonsynonymous mutations segregating in the DARC gene region in gorillas, chimpanzees, and bonobos.
(PDF)

S12 Table T_MRCA_ of *Plasmodium vivax* genes. (PDF)

S13 Table Summary statistics from ABC simulations. The summary statistics of the real FY*O data and the simulations from each of the five tested initial frequency models. The percentages represent the quantiles in the data (Ex. 50% is the median).
(XLSX)

S14 Table Summary statistics from ABC simulations for fixed selected alleles. The summary statistics of the real FY*O data and the simulations from each of the five tested initial frequency models where the selection allele fixed in the population. The percentages represent the quantiles in the data (Ex. 50% is the median).
(XLSX)

S15 Table Information gain statistics of features from 0.1% initial frequency simulation. (XLSX)
