# Supplementary material for: Population genetic analysis of the DARC locus (Duffy) reveals adaptation from standing variation associated with malaria resistance in humans
Source: PLoS Genet. 2017 Mar 10;13(3):e1006560. doi: 10.1371/journal.pgen.1006560 (PMC5365118; doi:10.1371/journal.pgen.1006560)
Supplement: S1 Appendix — Elaboration on the initial frequency & selection coefficient estimator. (PDF) [file pgen.1006560.s001.pdf]

1 Appendix

1.1 FY*O Initial Frequency and Selection Coefficient Estimations

FY*O's two divergent, common haplotypes in Africa indicate it may have reached fixation due to selection on standing variation. We infer that the FY*O mutation underwent a selective sweep on standing variation with a selection coefficient comparable to some of the most strongly selected loci in the human genome. Utilizing a Bayesian model selection approach implemented in an ABC framework, we find that FY*O likely rose to fixation via selection on standing variation; though the frequency of FY*O at selection onset was very low (0.1%).

Interestingly, we find that an FY*O initial frequency of magnitude 0.1% provides the best fit to our data. Among models of higher initial starting frequency (ex. 10% and 25%), the simulations which have resulted in the fixation of the selected allele have an excess of diversity (ex. average number of pairwise differences in 10% simulations is 3.98 (95% CI: 1.71 – 7.51) vs. 1.17 in the real data), while *de novo* mutations are very unlikely to fix and have a dearth of diversity when they do fix in the population (ex. average number of pairwise mutations in fixed *de novo* models is 0.18 (95% CI: 0.06 – 0.38) compared with the real data 1.17).

We conducted a variety of analyses to test the robustness of our inferences. First, we conducted cross validation for our model selection approach. To accomplish this, we randomly chose 50 simulations from each frequency model and utilized our ABC approach to infer their initial frequency model. We found some overlap between adjacent models, but high power when looking in the range of FY*O's inferred selection coefficient (below). We note, however, that in the 95% CI range of our estimated selection coefficient, we infer 85% of 0.1% frequency simulations correctly.

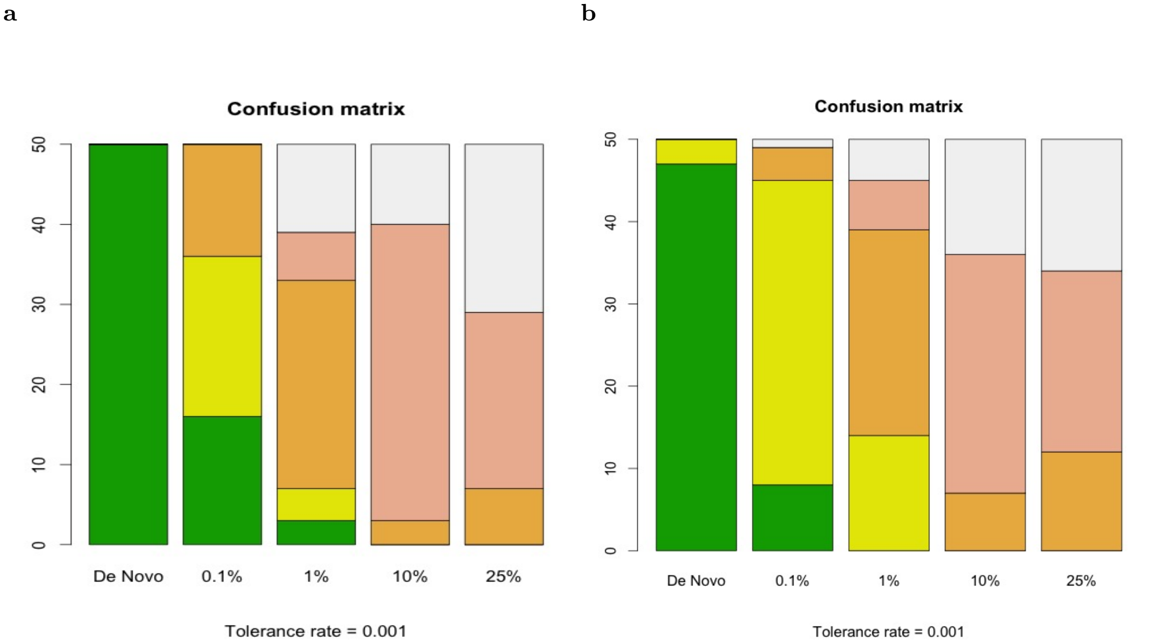

Figure 1. Cross validation of model selection Results from leave-out-one cross validation of the models. The x axis is the real model each simulation was drawn from, while the colors indicate the model it was assigned. Colors: Green, *de novo*; Yellow, 0.1%; Orange, 1%; Pink, 10%; Gray, 25%. a: For all simulation coefficient ranges. b: For simulations in the 95% CI of the estimated selection coefficient range.

We also verified that the 0.1% frequency model has a good fit to the data by comparing the observed data's place in the null distribution under each of the five models. For each model we limited to the 1% of simulations most similar to the observed data. Then, we randomly drew one simulation from that group and calculated the mean distance from the drawn simulation to all the others (based on the Euclidean distance of the PLS-DA transformed summary statistics). This procedure was repeated 100 times. The below histogram reports the mean distances for each simulation as well as the mean distance of the observed data from each simulation. We find the 0.1% frequency model is the only model where the observed value has a probability of being from the null distribution of greater than 0.05 ($p = 0.27$), supporting our model selection result.

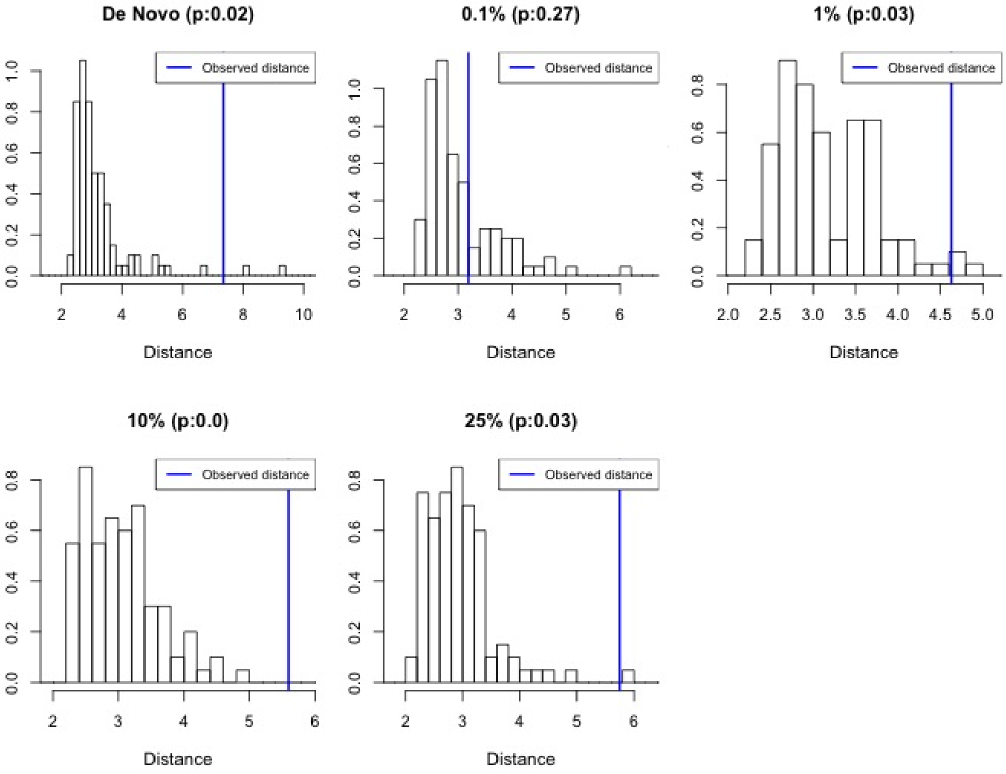

Figure 2. Observed value and null distribution under each model

We evaluated the effect of different tolerance rates, number of simulations, recombination rates, start times of selection, and demographic models. We find our results to be robust to a wide range of these parameters. Varying the tolerance rates and number of simulations results in the 0.1% model having between an 81% and 96% posterior probability.

Tolerance	# sims	Rejection	*De novo*	0.1%	1%	10%	25%
0.1%	100,000	*	0	0.920	0.080	0	0
0.1%	50,000	*	0.008	0.928	0.069	0	0
0.1%	10,000	*	0.020	0.960	0.020	0	0
0.5%	100,000		0.012	0.912	0.076	0	0
0.5%	50,000		0.018	0.929	0.070	0	0
0.5%	10,000	*	0.012	0.880	0.104	0.004	0
1%	100,000		0.0002	0.9167	0.0827	0.0004	0
1%	50,000		0.0002	0.9158	0.0838	0.0001	0
1%	10,000	*	0.072	0.818	0.108	0.002	0
5%	100,000		0.0012	0.9112	0.086	0.0014	0.0003
5%	50,000		0.0017	0.9105	0.0856	0.0018	0.0004
5%	10,000		0.0016	0.8983	0.0979	0.0017	0.0004

Table 1. Results by number of simulations and tolerance rate. * indicates that the rejection algorithm was used because some models had no accepted simulations.

Results varying the time since selection onset and the recombination rate show that the 0.1% model is the best-fit model for a wide range of parameters. It is only when we set the selection onset to 160 kya (4x greater than our inferred) that we find the 0.1% and 1% models have similar probabilities. We also inferred the recombination rate in the region. For this analysis, LDhat was

run for 40 million iterations with a block penalty of 5, sampling every 30,000 steps (Auton et al. 2012) on the FY*O background for regions of 20 Kb around the chromosome position for the duffy null mutation. For each run, Rmin values as per Hudson and Kaplan (1985) were estimated to have a point estimate recombination rate for the region. We estimate a lower recombination rate in the region than the deCODE project; however, we find our ABC model is robust to this lower recombination rate. When we test a different model of African demography (cartoon version of model from Li and Durbin 2011), we find the 0.1% and 1% frequency models to have similar probabilities. The *de novo* mutation model has a very low probability in all scenarios tested.

**Model**	**Time since sel.**	**Recom. rate (cM/MB)**	***De novo***	**0.1%**	**1%**	**10%**	**25%**
Gutenkunst et al.	20 kya	3.33	0.0003	0.983	0.0169	0	0
Gutenkunst et al.	60 kya	3.33	0.0001	0.885	0.115	0.001	0
Gutenkunst et al.	80 kya	3.33	0.0005	0.755	0.246	0.001	0
Gutenkunst et al.	160 kya	3.33	0	0.470	0.515	0.005	0.0102
Gutenkunst et al.**	40 kya	0.742	0.0006	0.9261	0.0719	0	0.0014
Gutenkunst et al.	40 kya	0.833 (0.25x)	0.0001	0.9123	0.0875	0	0.001
Gutenkunst et al.	40 kya	1.66 (0.5x)	0	0.927	0.0734	0	0
Gutenkunst et al.	40 kya	6.66 (2x)	0	0.955	0.0454	0	0
Gutenkunst et al.	40 kya	13.32 (4x)	0.0032	0.9785	0.0184	0	0
Li & Durbin*	40 kya	5	0.0020	0.4359	0.5178	0.0187	0.0256

Table 2. Results by time since selection onset and recombination rate. These results are based off of 10,000 simulations and a 1% acceptance rate. *This is a version of the Li & Durbin (2011) model, based off of the African portion of 'sim-split2' in their Supplementary Information **This recombination rate is based off of our inference of the recombination rate. The main recombination rate used in this paper was based off of estimates from the deCODE project.

We also report the Bayes factors for our model selection. This is based on 100,000 simulations per model, and a multinomial logistic regression model with a tolerance rate of 0.01. This assumed a recombination rate of 3.33 cM/MB and a selection start time of 40 kya.

	De novo	**0.1%**	**1%**	**10%**	**25%**
De novo	1	0.0002	0.0025	0.5041	51.04
**0.1%**	4377	1	11.09	2207	223417
**1%**	395	0.0902	1	199.1	20155
**10%**	1.98	0.0005	0.005	1	101.2
**25%**	0.0196	0	0	0.0099	1

Table 3. Bayes factors for model selection.

Next, we move to our second step: estimating the selection coefficient, based on the initial frequency model. We utilize leave-out-one cross validation with a tolerance rate of 0.1% to evaluate the accuracy of parameter inference. We also have reasonable power to infer the selection coefficient ($r^2 = 0.85$).

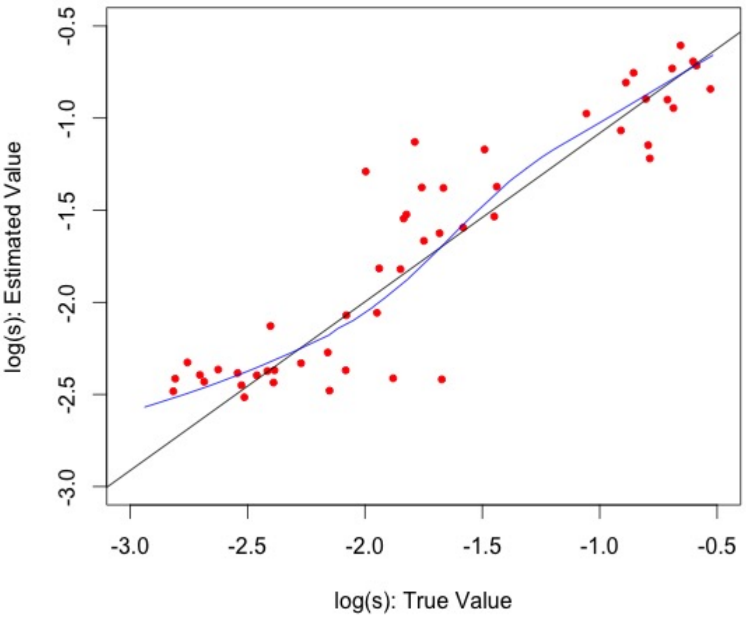

Figure 3. Cross-validation for parameter inference of log(s) for 0.1% initial frequency simulations We utilize leave-out-one cross validation with a tolerance rate of 0.1% to evaluate the accuracy of parameter inference. Black line: Linear best fit, Blue line: Lowess line of best fit, $r^2 = 0.848$

We also conducted a posterior predictive check. We sampled selection coefficients (s) from the posterior distribution of s, based on the 0.1% allele frequency at selection onset. We ran simulations with these selection coefficients and the initial frequency drawn from either $10^{U(-5,-0.00001)}$ or $U(0,1)$. Then, we used local linear regression to infer the initial frequency of our data. With the log-based prior distribution, we find high correlation between the simulated and inferred initial frequencies ($r^2 = 0.84$), and we re-estimate the initial frequency at 0.15% (95% CI: 0.018 – 0.77%; Fig 4), closely fitting our inferred best fit model. However with the uniform prior distribution, we have much lower power to estimate initial allele frequency. We find poor correlation between the simulated and inferred initial frequencies ($r^2 = 0.076$) and we re-estimate the initial frequency at 6.86% (95% CI: -20.3 – 51.6%, Fig 5). This is a very wide (and biologically impossible) confidence interval. This is not surprising, as it has previously been shown that it is very difficult to estimate initial frequency with this prior (Peter et al. 2012).

a **b**

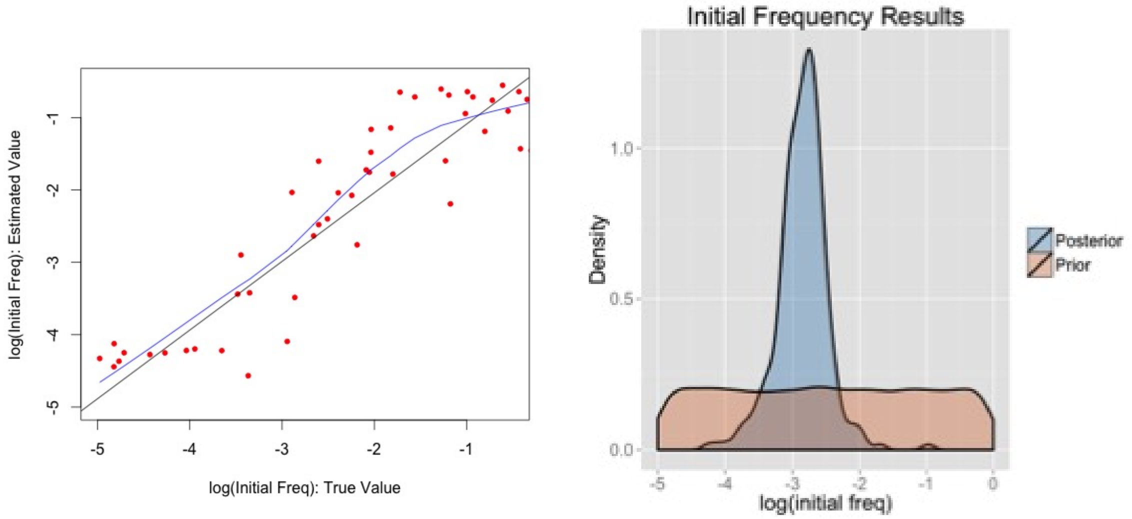

Figure 4. Posterior check: Cross-validation for initial frequency $10^{U(-5,-0.00001)}$ **prior** a: True initial frequency vs. inferred initial frequency; Black line: Linear best fit, Blue line: Lowess line of best fit. b: Prior and posterior distributions of the initial frequency.

a **b**

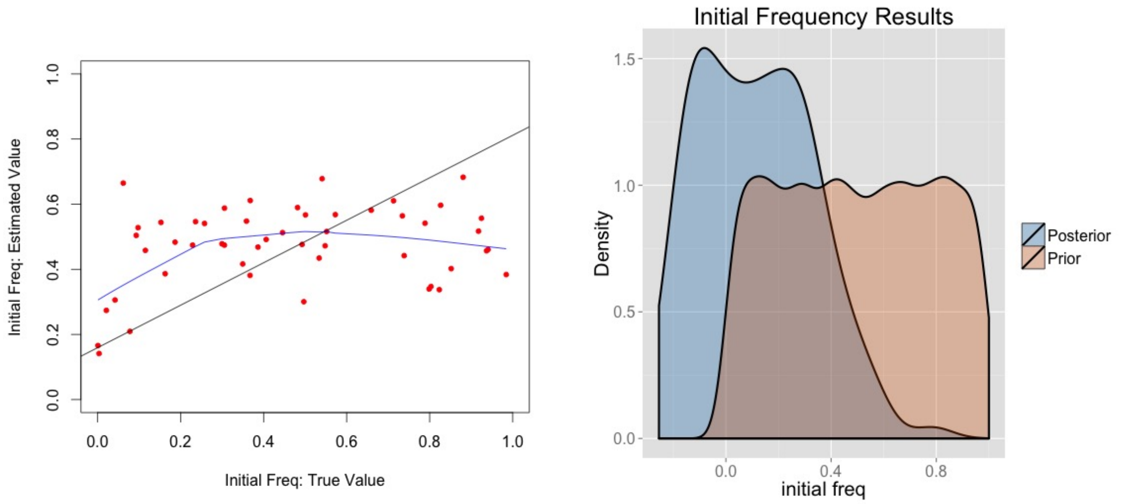

Figure 5. Posterior check: Cross-validation for initial frequency with U(0,1) prior a: True initial frequency vs. inferred initial frequency; Black line: Linear best fit, Blue line: Lowess line of best fit. b: Prior and posterior distributions of the initial frequency.

We conducted a second posterior predictive check for the uniform and log priors using a restricted initial frequency range of 0.01% to 10%. Similar to before, we find low correlation with the uniform prior ($r^2 = 0.27$), and reestimate the frequency at -0.01% (95% CI: -2.0 – 4.0%, Fig 7). With the log prior we find ($r^2 = 0.89$) and reestimate the frequency at 0.098% (95% CI: 0.0062 – 0.40 %, Fig 6).

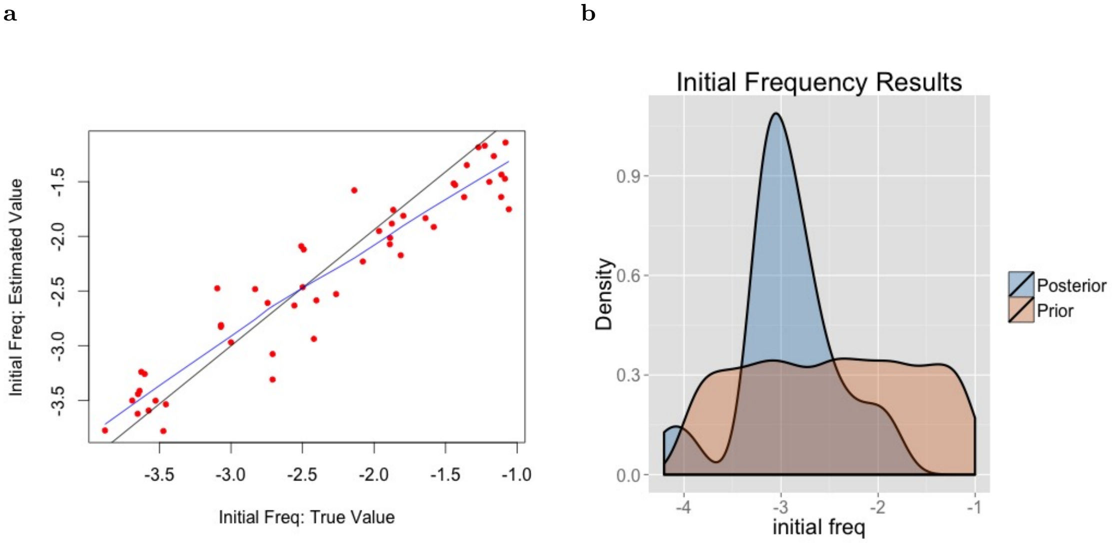

Figure 6. Posterior check: Cross-validation for initial frequency with log(0.0001,0.1) prior a: True initial frequency vs. inferred initial frequency; Black line: Linear best fit, Blue line: Lowess line of best fit. b: Prior and posterior distributions of the initial frequency.

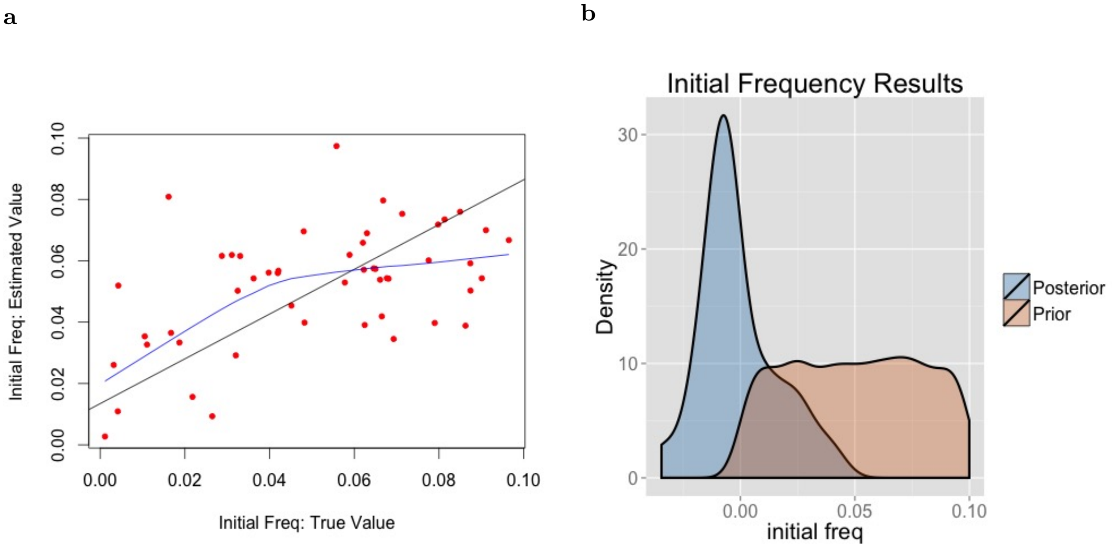

Figure 7. Posterior check: Cross-validation for initial frequency with U(0.0001,0.1) prior a: True initial frequency vs. inferred initial frequency; Black line: Linear best fit, Blue line: Lowess line of best fit. b: Prior and posterior distributions of the initial frequency.

We investigate how varying the time since selection and the recombination rate affect the selection coefficient inference. In this analysis, we use 10,000 simulations for each case and a 1% acceptance rate. For each case, we use the best fit initial frequency model from the previous analysis, which is 0.1% in all cases except for the model with selection starting 160 kya and the Li and Durbin (2011) demographic model. The inferred selection coefficients are all between 0.025 - 0.058, besides the 1% frequency model which has a lower inferred s (0.0072) with a 160 kya selection start time and a higher inferred s (0.1151) with the Li and Durbin (2011) model. As we increase the recombination rate or increase the time since selection, we note decreasing inferred selection coefficients. However, they are still in the range of our inferred value.

Model	Time since sel.	Recom rate (cM/MB)	Init freq	Selection coeff
Gutenkunst et al.	20 kya	3.33	0.1%	0.0584 (95% CI: 0.0223 - 0.1885)
Gutenkunst et al.	60 kya	3.33	0.1%	0.0327 (95% CI: 0.0134 - 0.0893)
Gutenkunst et al.	80 kya	3.33	0.1%	0.049 (95% CI: 0.0121 - 0.186)
Gutenkunst et al.	160 kya	3.33	0.1%	0.0278 (95% CI : 0.0162 - 0.0400)
Gutenkunst et al.	160 kya	3.33	1%	0.0072 (95% CI: 0.0032-0.0072)
Gutenkunst et al.**	40 kya	0.742	0.1%	0.0551 (95% CI: 0.0164 - 0.201)
Gutenkunst et al.	40 kya	0.833 (0.25x)	0.1%	0.0532 (95% CI: 0.0125 - 0.400)
Gutenkunst et al.	40 kya	1.66 (0.5x)	0.1%	0.0504 (95% CI: 0.0113 - 0.213)
Gutenkunst et al.	40 kya	6.66 (2x)	0.1%	0.0420 (95% CI: 0.0106 - 0.1371)
Gutenkunst et al.	40 kya	13.32 (4x)	0.1%	0.0409 (95% CI: 0.0010 - 0.1661)
Li & Durbin*	40 kya	5	0.1%	0.1151 (95% CI: 0.0316 - 0.3899)
Li & Durbin*	40 kya	5	1%	0.0250 (95% CI: 0.0129 - 0.0800)

Table 4. ABC selection coefficient results when varying time since selection onset and recombination rate. These results are based off of 10,000 simulations and a 1% acceptance rate. *This is a version of the Li & Durbin (2011) model, based off of the African portion of 'sim-split2' in their Supplementary Information **This recombination rate is based off of our inference of the recombination rate in the 20 KB region surrounding FY*O in African sample in the 1000 Genome dataset that are homozygous for FY*O. The main recombination rate used in this paper was based off of estimates from the deCODE project.

We also include a figure showing the inferred selection coefficient assuming a model of selection on *de novo* mutation, which was not a good fit to our data. Unlike the figure for selection on allele with 0.1% frequency, selection on *de novo* mutation results in a posterior distribution much wider than its prior distribution. It inferred a selection coefficient of 0.006428 (95% CI: 3.19e-37 - 1.57e9).

a **b**

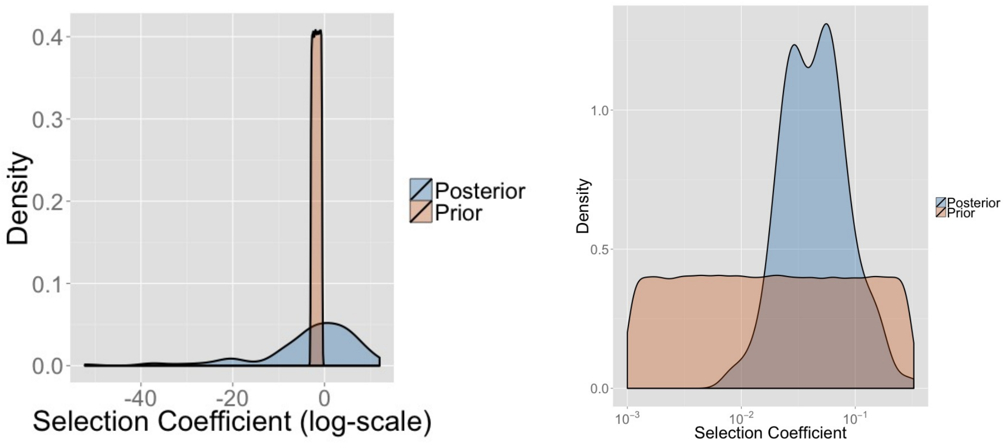

Figure 8. a: Prior and posterior density of selection coefficient assuming *de novo* mutation b: Prior and posterior density of selection coefficient assuming 0.1% initial frequency (same as Figure 3 in the main paper).

Auton A, Fledel-Alon A, Pfeifer S, Venn O, Segurel L, Street T, Leffler EM, Bowden R, Aneas I, Broxholme J, et al. 2012. A fine-scale chimpanzee genetic map from population sequencing. Science 336:193–198.

Hudson RR and Kaplan N. 1985. Statistical properties of the number of re- combination events in the history of a sample of DNA sequences. Genetics, 111:147–164.

1.2 Data Processing

SNPs used in this analysis were called via two methods. The high-coverage 1000 Genomes samples were called as described in [?], while the other low-coverage populations were called as described in the methods. The 1000 Genomes samples have very high quality called SNPs, and have integrated high coverage exome data into their results. Therefore, we didn't want to recall these high quality samples with our other low coverage samples. The low coverage samples were used because they included some important population samples (ex. the San and the hunter gatherers). We note there is likely calling bias between the 1000 Genomes integrated dataset and the low coverage samples recalled in this paper. This is evidenced by multiple SNPs being present only in the recalled low-coverage data (Fig 2B), despite some populations in the recalled data being highly divergent from each other but close to those in the 1000 genomes data. For example, the haplotype network (Fig 2B) contain a number of rare haplotypes exclusively shared by Bagandan (pink nodes) and Zulu (gray nodes) populations, two genetically more distant populations with low-coverage genotype calls. This contrasts with less shared rare haplotypes of the Bagandan with LWK (from the 1000 Genomes dataset), which are genetically and geographically closer than the Zulu [?]. As a consequence, we conducted the bulk of the analyses separately by population. Our results show that this calling bias does not affect our conclusions. For example, T_{MRCA} estimates of 1000 Genomes populations and recalled samples are very similar (S8-S10 Tables). The ABC analysis was conducted on the LWK population, which is part of the higher quality 1000 Genomes sample set.
