# Supplementary figures and images for: Population genetic analysis of the DARC locus (Duffy) reveals adaptation from standing variation associated with malaria resistance in humans
Source: PLoS Genet. 2017 Mar 10;13(3):e1006560. doi: 10.1371/journal.pgen.1006560 (PMC5365118; doi:10.1371/journal.pgen.1006560)

a.

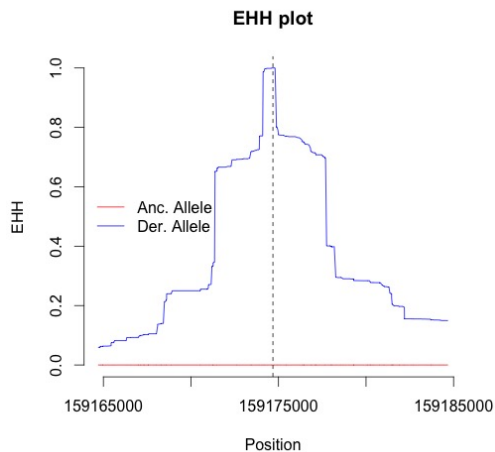

b.

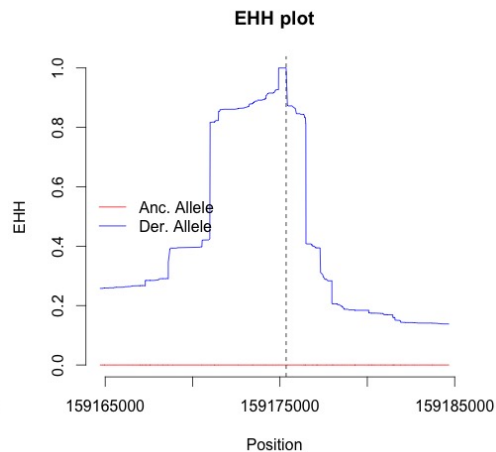

c.

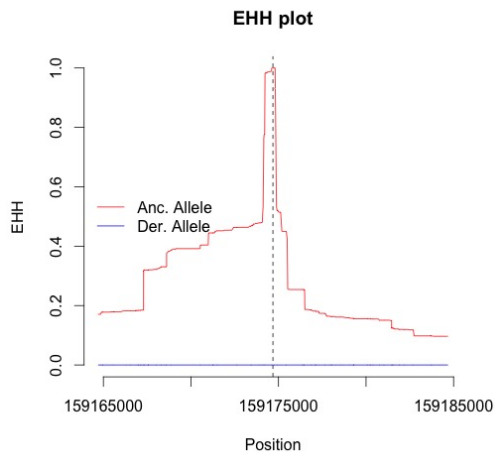

d.

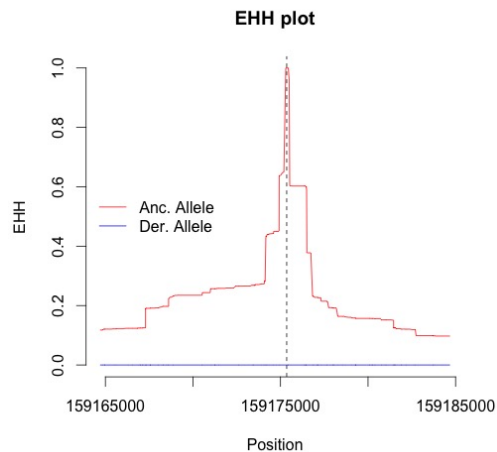

Supplement: S1 Fig — EHH plots for the 20kb region surrounding the FY*O mutation. A) FY*O samples centered on FY*O mutation B) FY*A samples centered on FY*A mutation C) FY*B samples centered on FY*O mutation D) FY*B samples centered on FY*A mutation. (PDF) [file pgen.1006560.s002.pdf]

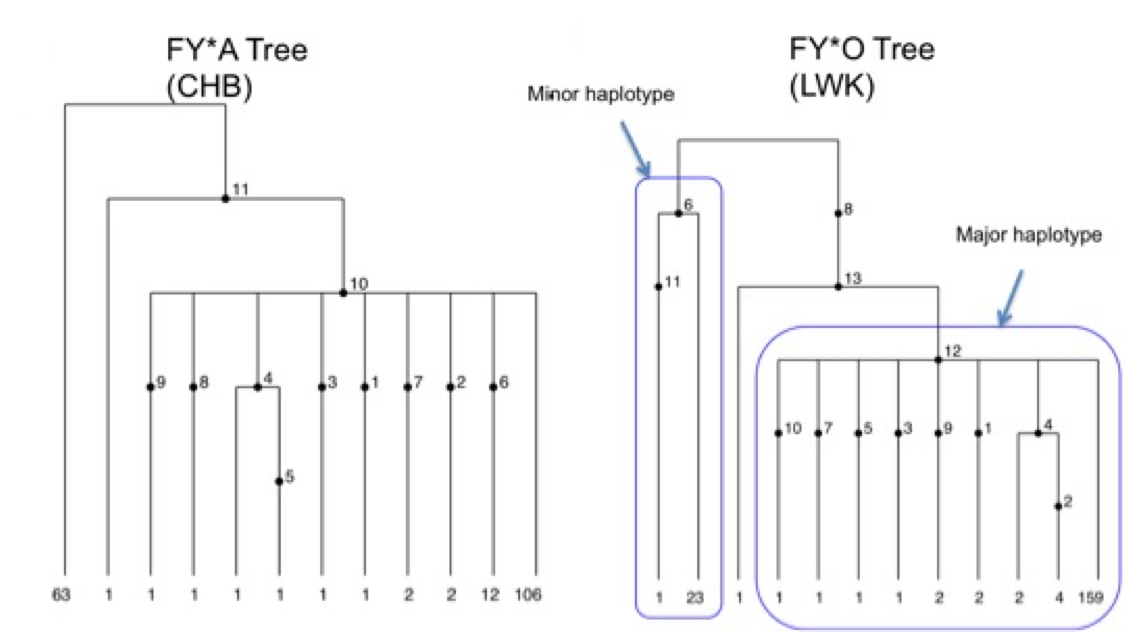

Supplement: S2 Fig — Geneology from Genetree of the 5kb region around FY*O. Dots indicate mutations and bottom numbers indicate number of samples with that haplotype. Left: geneology of FY*A samples from CHB population. Right: geneology of FY*O samples from LWK population. (PNG) [file pgen.1006560.s003.png]

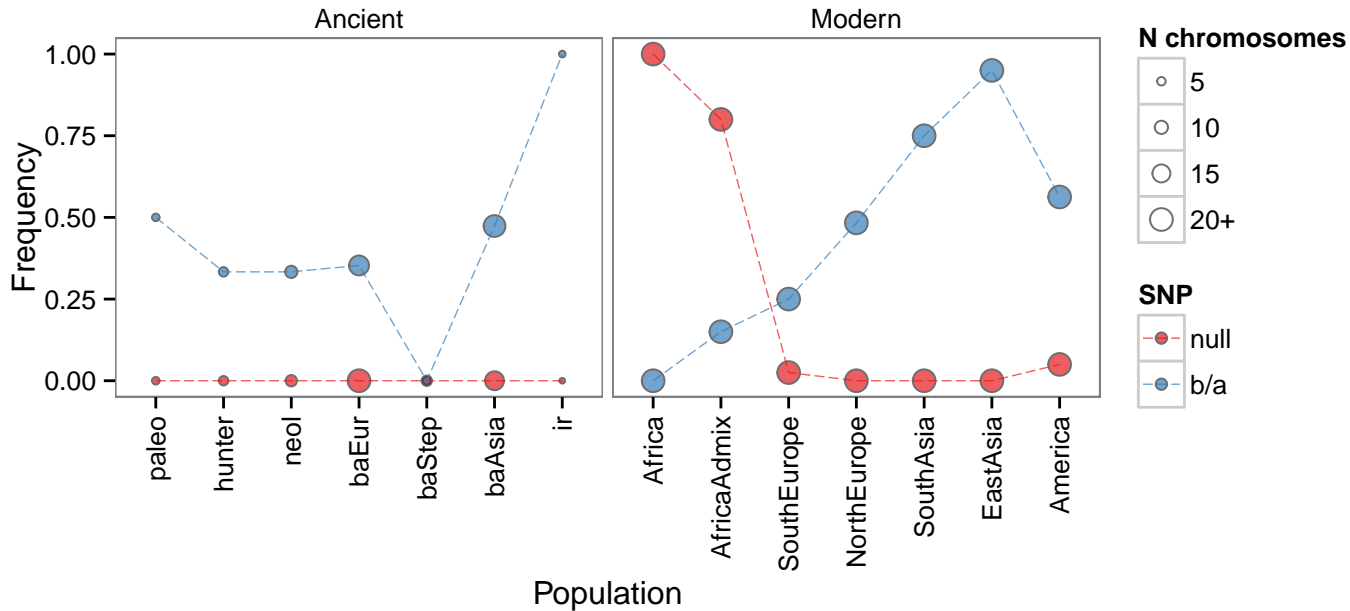

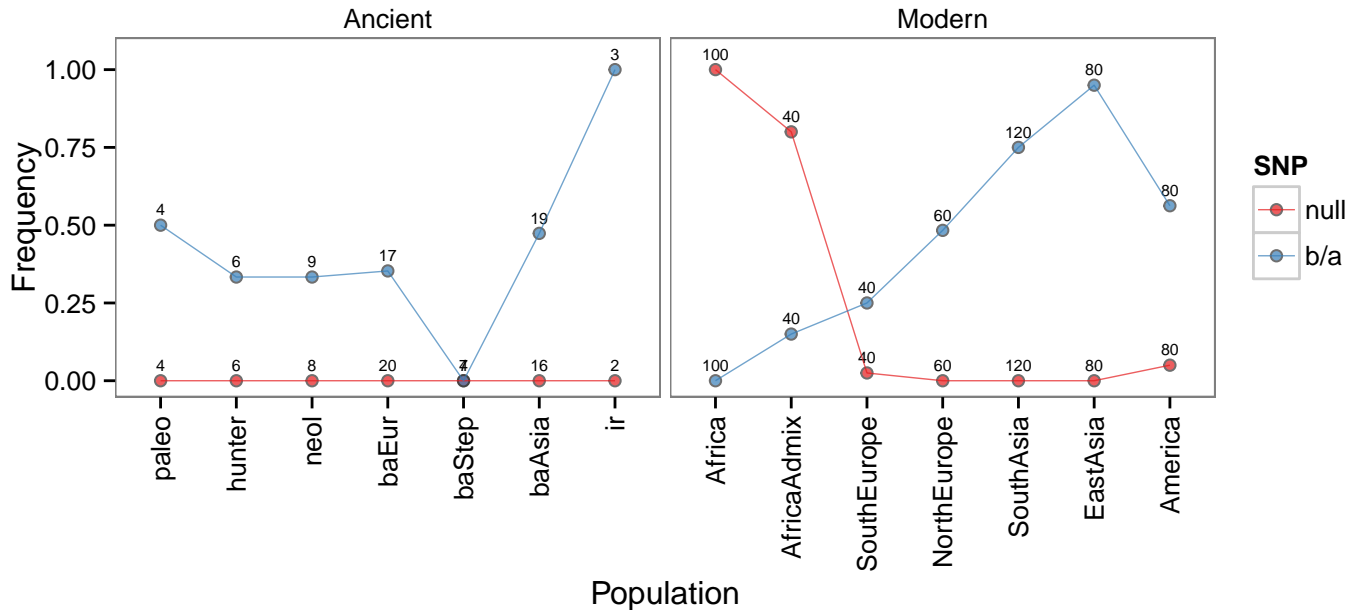

Supplement: S3 Fig — Paleo: paleolithic; Hunter: hunter-gatherer; neol: neolithic; baEur: Bronze Age Europe; baStep: Bronze Age Steppe region; baAsia: Bronze Age Asia; ir: Iron Age. Sequences from Allentoft et al. (2015) [56]. (PDF) [file pgen.1006560.s004.pdf]

a.

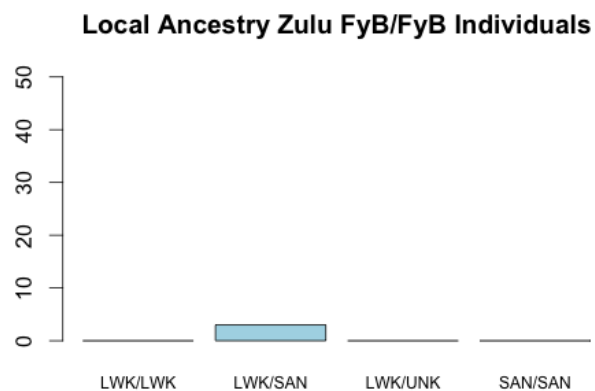

b.

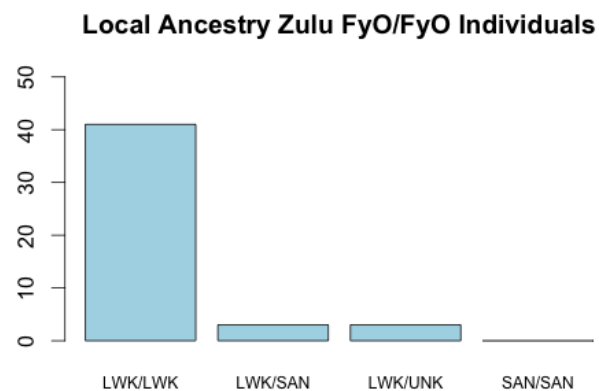

c.

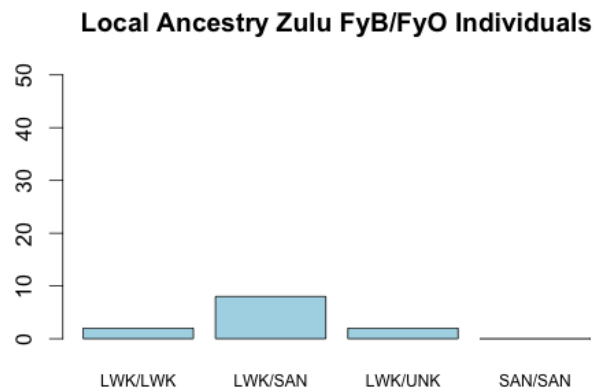

d.

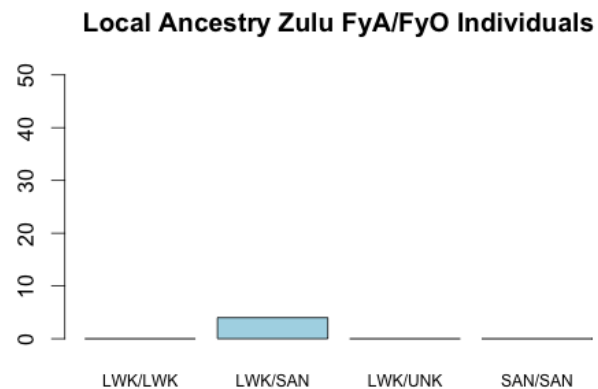

Supplement: S5 Fig — There were no homozygous FY*A samples. A) Homozygous FY*B samples B) Homozygous FY*O samples C) FY*B/FY*O samples D) FY*A/FY*O samples. (PDF) [file pgen.1006560.s006.pdf]

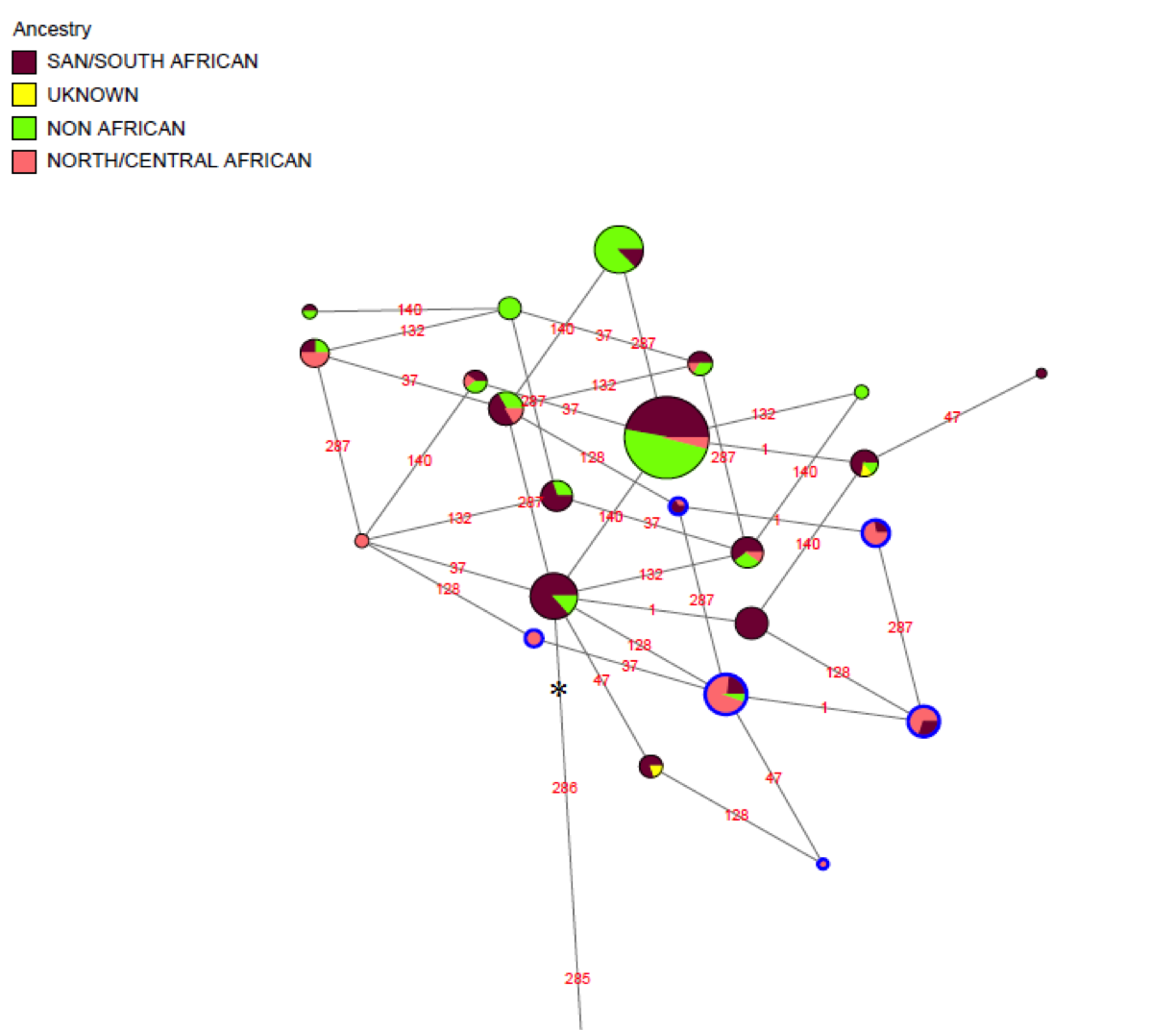

Supplement: S6 Fig — Weights are based on GERP conservation score. Asterisk indicates the root of the network. Blue circles indicate FY*O haplotypes. (PNG) [file pgen.1006560.s007.png]

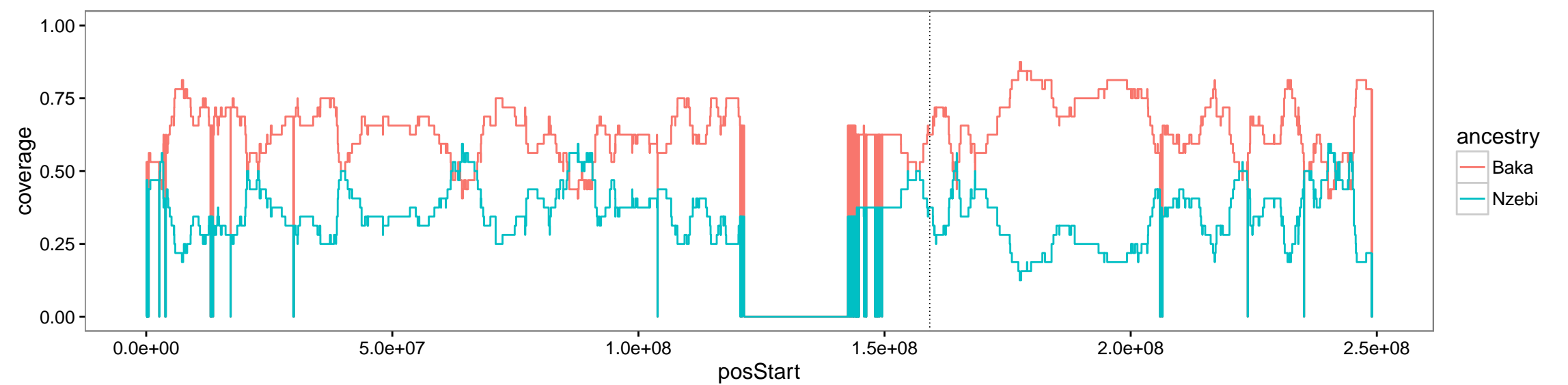

Supplement: S7 Fig — Dashed line indicates position of the DARC gene. Local ancestry in Baka Pygmies was inferred using RFMix [115]). Due to the unavailability of an unadmixed source population panel for the Pygmy ancestry, we initially ran RFMix on a single Baka individual as target with the remaining individuals as source population. Local ancestry was then updated in both the Baka and Nzebi sources using four EM iterations. (PDF) [file pgen.1006560.s008.pdf]
