# Supplementary material for: Population genetic analysis of the DARC locus (Duffy) reveals adaptation from standing variation associated with malaria resistance in humans
Source: PLoS Genet. 2017 Mar 10;13(3):e1006560. doi: 10.1371/journal.pgen.1006560 (PMC5365118; doi:10.1371/journal.pgen.1006560)
Supplement: S4 Fig — A) Homozygous FY*B samples B) Homozygous FY*O samples C) Homozygous FY*A samples D) FY*O/FY*B samples E) FY*A/FY*B samples F) FY*A/FY*O samples. (PDF) [file pgen.1006560.s005.pdf]

a

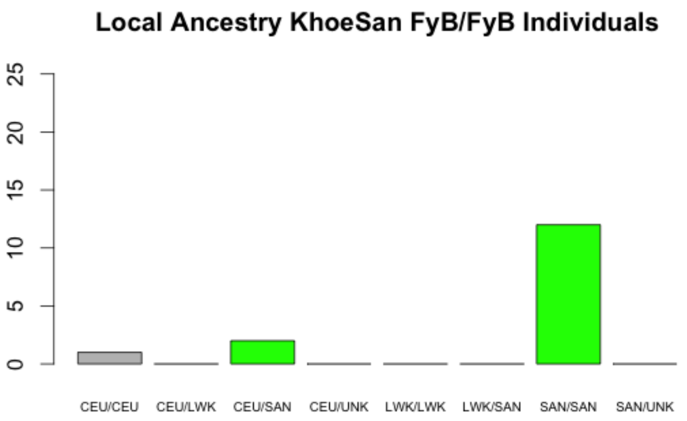
Local Ancestry KhoeSan FyB/FyB Individuals
0
5
10
15
20
25
CEU/CEU
CEU/LWK
CEU/SAN
CEU/UNK
LWK/LWK
LWK/SAN
SAN/SAN
SAN/UNK

b

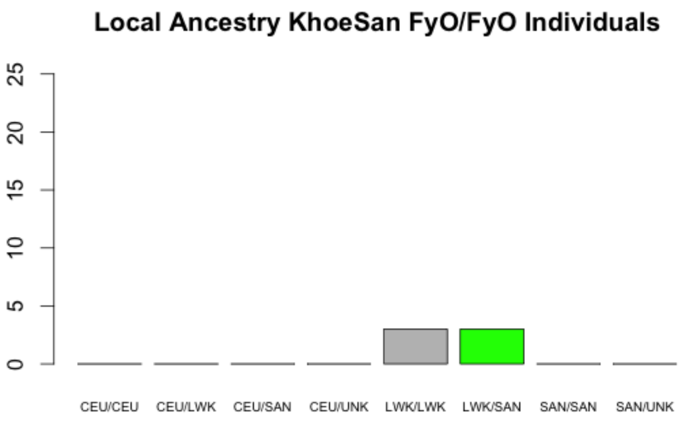
Local Ancestry KhoeSan FyO/FyO Individuals
0
5
10
15
20
25
CEU/CEU
CEU/LWK
CEU/SAN
CEU/UNK
LWK/LWK
LWK/SAN
SAN/SAN
SAN/UNK

c

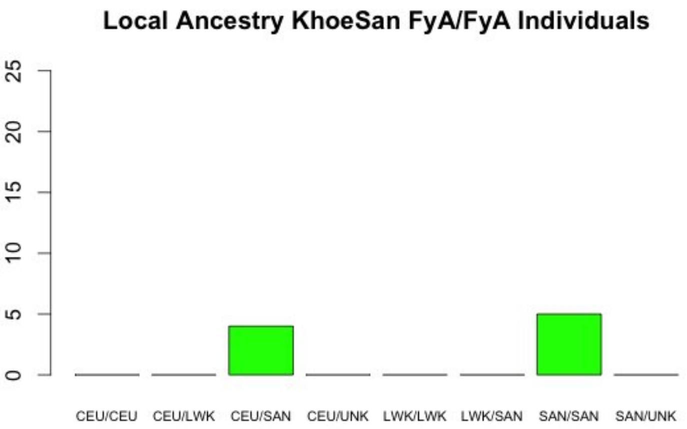
Local Ancestry KhoeSan FyA/FyA Individuals
0
5
10
15
20
25
CEU/CEU
CEU/LWK
CEU/SAN
CEU/UNK
LWK/LWK
LWK/SAN
SAN/SAN
SAN/UNK

d

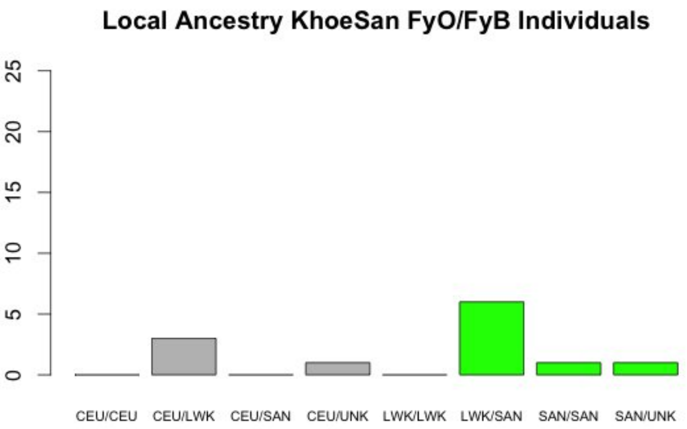
Local Ancestry KhoeSan FyO/FyB Individuals
0
5
10
15
20
25
CEU/CEU
CEU/LWK
CEU/SAN
CEU/UNK
LWK/LWK
LWK/SAN
SAN/SAN
SAN/UNK

e

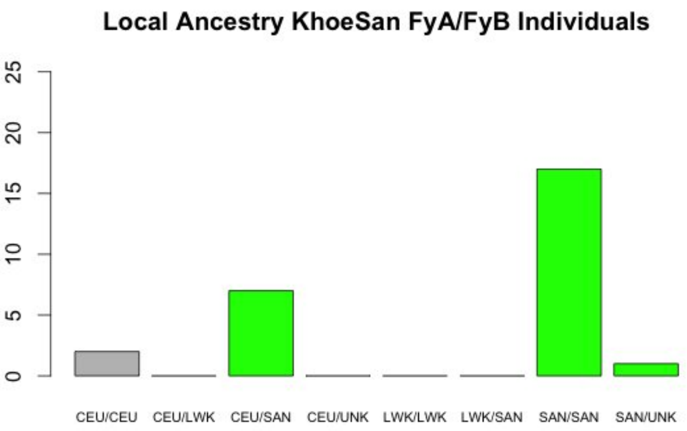
Local Ancestry KhoeSan FyA/FyB Individuals
0
5
10
15
20
25
CEU/CEU
CEU/LWK
CEU/SAN
CEU/UNK
LWK/LWK
LWK/SAN
SAN/SAN
SAN/UNK

f

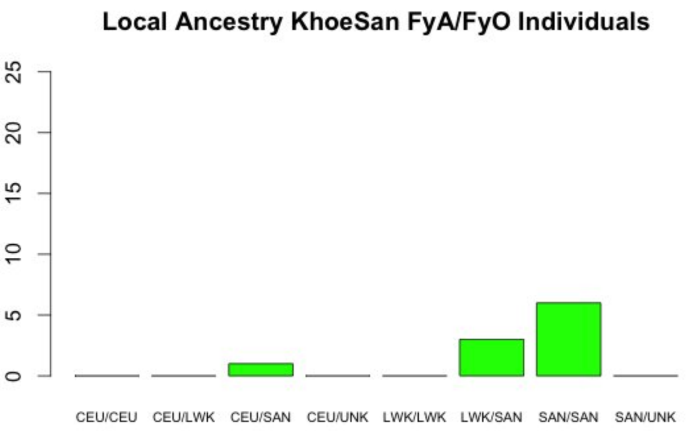
Local Ancestry KhoeSan FyA/FyO Individuals
0
5
10
15
20
25
CEU/CEU
CEU/LWK
CEU/SAN
CEU/UNK
LWK/LWK
LWK/SAN
SAN/SAN
SAN/UNK
