# Supplementary material for: Population genetic analysis of the DARC locus (Duffy) reveals adaptation from standing variation associated with malaria resistance in humans
Source: PLoS Genet. 2017 Mar 10;13(3):e1006560. doi: 10.1371/journal.pgen.1006560 (PMC5365118; doi:10.1371/journal.pgen.1006560)
Supplement: S1 Table — (PDF) [file pgen.1006560.s009.pdf]

Continent	Population	Location	Dataset	Sample number
Africa	Luhya (LWK)	Kenya	1000G	99
	Yoruba (YRI)	Nigeria	1000G	108
	Esan (ESN)	Nigeria	1000G	99
	Gambian (GWD)	the Gambia	1000G	113
	Mende (MSL)	Sierra Leone	1000G	85
	Baganda	Uganda	AGVP	100
	Zulu	South Africa	AGVP	100
	Baka	Gabon	In-house	20
	Nzebi	Gabon	In-house	20
	Mbuti	DR Congo	HGDP	7
	≠Khomani San	South Africa		
Europe	Utah Residents with Northern & Western European Ancestry (CEU)	Utah	1000G	99
	Finnish (FIN)	Finland	1000G	99
	British (GBR)	UK	1000G	91
	Iberian (IBS)	Spain	1000G	107
	Toscani (TSI)	Italy	1000G	107
Asian	Chinese Dai (CDX)	China	1000G	93
	Han Chinese (CHB)	China	1000G	103
	Southern Han Chinese (CHS)	China	1000G	105
	Japanese (JPT)	Japan	1000G	104
	Kinh (KHV)	Vietnam	1000G	99
