# Supplementary material for: Population genetic analysis of the DARC locus (Duffy) reveals adaptation from standing variation associated with malaria resistance in humans
Source: PLoS Genet. 2017 Mar 10;13(3):e1006560. doi: 10.1371/journal.pgen.1006560 (PMC5365118; doi:10.1371/journal.pgen.1006560)
Supplement: S2 Table — (PDF) [file pgen.1006560.s010.pdf]

Population	FY*O						FY*A				
	# Indiv	T	C	T/T	T/C	C/C	G	A	G/G	G/A	A/A
African											
**YRI**	108	1	215	0	1	107	0	216	0	0	108
**LWK**	99	0	198	0	0	99	0	198	0	0	99
**ESN**	99	0	198	0	0	99	0	198	0	0	99
**GWD**	113	0	226	0	0	113	0	226	0	0	113
**MSL**	85	0	170	0	0	85	0	170	0	0	85
**Baganda**	100	0	200	0	0	100	1	199	0	1	99
**Zulu**	100	43	157	9	25	66	14	186	1	12	87
**Baka**	20	0	40	0	0	20	0	40	0	0	20
**Nzebi**	20	0	40	0	0	20	0	40	0	0	20
**Mbuti**	7	0	14	0	0	7	0	14	0	0	7
**≠Khomani San**	83	130	36	55	22	6	63	103	10	39	34
European											
**CEU**	99	198	0	99	0	0	85	113	16	53	30
**FIN**	99	198	0	99	0	0	90	108	24	42	33
**GBR**	91	182	0	91	0	0	76	106	9	58	24
**IBS**	107	210	4	103	4	0	64	150	10	44	53
**TSI**	107	212	2	105	2	0	85	129	16	53	38
Asian											
**CDX**	93	186	0	93	0	0	177	9	84	9	0
**CHB**	103	206	0	103	0	0	192	14	90	12	1
**CHS**	105	210	0	105	0	0	196	14	91	14	0
**JPT**	104	208	0	104	0	0	185	23	82	21	1
**KHV**	99	198	0	99	0	0	189	18	81	18	0
