# Supplementary material for: Population genetic analysis of the DARC locus (Duffy) reveals adaptation from standing variation associated with malaria resistance in humans
Source: PLoS Genet. 2017 Mar 10;13(3):e1006560. doi: 10.1371/journal.pgen.1006560 (PMC5365118; doi:10.1371/journal.pgen.1006560)
Supplement: S3 Table — Nucleotide diversity statistics in the 5kb, 10kb, and 20kb region surrounding the FY*O mutation. (PDF) [file pgen.1006560.s011.pdf]

	5 kb				10 kb			20 kb		
	# Indiv.	S	π	Tajima's D	S	π	Tajima's D	S	π	Tajima's D
African										
**YRI**	108	20	1.00	-1.87	49	5.73	-0.90	106	16.89	-0.16
**LWK**	101	14	1.17	-1.28	45	6.17	-0.58	108	18.74	0.05
**ESN**	99	14	0.72	-1.75	44	5.39	-0.83	100	16.25	-0.15
**GWD**	113	17	0.84	-1.81	45	5.41	-0.81	108	17.16	-0.15
**MSL**	85	14	1.23	-1.28	40	5.75	-0.53	102	17.37	-0.09
**Baganda**	100	17	1.65	-1.12	43	4.94	-0.96	104	17.52	-0.03
**Zulu**	100	29	3.05	-1.08	55	6.36	-0.96	125	18.62	-0.39
**Baka**	20	7	1.17	-0.80	17	3.58	-0.34	61	15.01	0.17
**Nzebi**	20	8	0.98	-1.37	15	3.20	-0.30	52	13.63	0.41
**Mbuti**	7	5	2.07	1.09	16	5.21	0.15	48	17.89	0.81
European										
**CEU**	91	30	5.40	0.16	65	9.44	-0.45	122	16.94	-0.58
**FIN**	98	30	5.35	0.13	54	9.36	0.05	94	17.01	0.19
**GBR**	92	34	5.57	-0.16	59	9.60	-0.18	99	17.51	0.07
**IBS**	106	38	6.15	-0.11	75	10.60	-0.49	144	19.39	-0.62
**TSI**	95	35	6.00	0.05	69	10.29	-0.34	142	18.67	-0.68
Asian										
**CDX**	93	20	2.32	-0.88	42	6.48	-0.31	76	12.45	-0.15
**CHB**	103	21	2.31	-0.95	40	6.15	-0.27	79	11.93	-0.33
**CHS**	105	24	2.21	-1.25	41	6.05	-0.37	72	11.73	-0.11
**JPT**	104	23	2.51	-0.96	40	6.21	-0.24	74	11.90	-0.15
**KHV**	99	21	2.64	-0.71	40	6.77	-0.02	77	12.92	-0.05
