# Supplementary material for: Population genetic analysis of the DARC locus (Duffy) reveals adaptation from standing variation associated with malaria resistance in humans
Source: PLoS Genet. 2017 Mar 10;13(3):e1006560. doi: 10.1371/journal.pgen.1006560 (PMC5365118; doi:10.1371/journal.pgen.1006560)
Supplement: S4 Table — Summary statistics were calculated in 750 bp region upstream from DARC and compared to the 750 bp region upstream from all other genes in genome in each population. Summary statistics calculated: number of segregating sites (s), number of pairwise differences (π), and Tajima’s D. We quantified the percentile in the genome (Per.), median, and 95% confidence interval (CI). (PDF) [file pgen.1006560.s012.pdf]

	S	per.	median	95% CI	π	per.	median	95% CI	Tajima's D	per.	median	95% CI
African												
**YRI**	3	28	5	0 – 14	0.20	21	0.59	0 – 2.6	-0.95	29	-0.44	-1.7 – 1.6
**LWK**	1	7	5	0 – 14	0.21	22	0.59	0 – 2.6	0.00	77	-0.55	-1.7 – 1.5
**ESN**	2	17	5	0 – 13	0.16	18	0.59	0 – 2.6	-0.73	37	-0.41	-1.6 – 1.6
**GWD**	2	16	5	0 – 14	0.16	23	0.59	0 – 2.6	-0.68	51	-0.52	-1.7 – 1.5
**MSL**	2	16	5	0 – 14	0.23	17	0.60	0 – 2.6	-0.48	42	-0.49	-1.7 – 1.5
European												
**CEU**	5	83	3	0 – 10	0.40	48	0.42	0 – 2.3	-1.02	18	0.00	-1.5 – 2.3
**FIN**	5	97	3	0 – 9	0.45	52	0.42	0 – 2.3	-1.51	1	0.00	-1.4 – 2.4
**GBR**	8	85	3	0 – 9	0.34	45	0.42	0 – 2.3	-1.15	13	0.00	-1.6 – 2.1
**IBS**	8	95	3	0 – 10	0.72	70	0.43	0 – 2.3	-1.02	23	0.00	-1.6 – 2.1
**TSI**	6	53	3	0 – 10	0.66	35	0.43	0 – 2.3	-0.71	24	0.00	-1.6 – 2.1
Asian												
**CDX**	3	28	3	0 – 9	0.07	39	0.39	0 – 2.2	-1.39	67	0.00	-1.5 – 2.4
**CHB**	4	62	3	0 – 9	0.09	39	0.39	0 – 2.2	-1.50	21	0.00	-1.6 – 2.3
**CHS**	6	65	3	0 – 9	0.10	38	0.38	0 – 2.2	-1.50	19	0.00	-1.5 – 2.4
**JPT**	3	48	3	0 – 9	0.04	36	0.38	0 – 2.2	-1.47	23	0.00	-1.5 – 2.4
**KHV**	2	43	3	0 – 9	0.10	40	0.39	0 – 2.2	-0.97	32	0.00	-1.6 – 2.3
