# Supplementary material for: Population genetic analysis of the DARC locus (Duffy) reveals adaptation from standing variation associated with malaria resistance in humans
Source: PLoS Genet. 2017 Mar 10;13(3):e1006560. doi: 10.1371/journal.pgen.1006560 (PMC5365118; doi:10.1371/journal.pgen.1006560)
Supplement: S5 Table — Weir and Cockerham’s weighted FST was calculated for each SNP in the genome and for 5 kb, 10 kb, and 20 kb windows. FST result and its percentile in the genome is reported for all fifteen 1000 Genomes populations. (PDF) [file pgen.1006560.s013.pdf]

	**FY*O F_{ST}**	**percentile**	**FY*A F_{ST}**	**percentile**
**SNP**	0.99	100	0.60	99.9
**5 kb region**	0.38	99.8	0.36	99.8
**10 kb region**	0.32	99.6	0.34	99.8
**20 kb region**	0.25	98.6	0.26	98.8
