# Supplementary material for: Population genetic analysis of the DARC locus (Duffy) reveals adaptation from standing variation associated with malaria resistance in humans
Source: PLoS Genet. 2017 Mar 10;13(3):e1006560. doi: 10.1371/journal.pgen.1006560 (PMC5365118; doi:10.1371/journal.pgen.1006560)
Supplement: S6 Table — We report the likelihood that the Duffy region (20 kb and 100 kb) underwent a recent hard selective sweep [52, 53]. This likelihood is determined via a composite likelihood ratio test where the numerator is the likelihood of the region site frequency spectrum given a hard selective sweep and the denominator is the likelihood of the region given a neutral model. This likelihood is compared to likelihoods from all other regions in the genome, as well as regions with average recombination rates within 25% of the Duffy region’s recombination rate. (PDF) [file pgen.1006560.s014.pdf]

	**20 kb**			**100 kb**		
	Likelihood	Percentile	Percentile (Recom. adj.)	Likelihood	Percentile	Percentile (Recom. adj.)
African						
**YRI**	4.36	95.8	97.5	8.37	98.1	99.4
**LWK**	2.96	93.0	95.1	2.29	90.2	92.3
**ESN**	3.07	93.3	95.3	5.14	96.6	97.8
**GWD**	2.71	92.3	94.3	5.50	97.0	98.4
**MSL**	2.13	89.1	91.6	3.26	89.5	96.0
European						
**CEU**	0.00	9.9	8.2	0.00	8.0	8.6
**FIN**	0.07	27.7	25.9	0.24	46.9	46.9
**GBR**	0.21	46.9	45.8	0.10	36.2	34.4
**IBS**	0.52	64.2	63.3	0.16	43.0	43.8
**TSI**	0.60	67.2	65.7	0.30	54.6	53.5
Asian						
**CDX**	2.50	87.4	87.2	4.39	92.2	92.2
**CHB**	2.23	86.5	86.6	3.68	91.5	90.9
**CHS**	0.00	13.9	13.0	2.03	83.8	84.9
**JPT**	1.60	80.7	80.9	0.49	60.9	61.1
**KHV**	1.37	80.3	80.8	4.74	93.0	93.2
