# Supplementary material for: Population genetic analysis of the DARC locus (Duffy) reveals adaptation from standing variation associated with malaria resistance in humans
Source: PLoS Genet. 2017 Mar 10;13(3):e1006560. doi: 10.1371/journal.pgen.1006560 (PMC5365118; doi:10.1371/journal.pgen.1006560)
Supplement: S7 Table — We report the maximum H-scan score for the Duffy region (20 kb and 100 kb). This score is then compared to the max score from all other regions in the genome, as well as regions with average recombination rates within 25% of the Duffy region’s recombination rate. (PDF) [file pgen.1006560.s015.pdf]

	**20 kb**			**100 kb**		
	Max Score	Percentile	Percentile (Recom. adj.)	Max Score	Percentile	Percentile (Recom. adj.)
African						
**YRI**	12148	82.7	84.2	16488	85.2	87.4
**LWK**	11454	75.7	87.0	13218	80.8	91.8
**ESN**	14450	91.1	96.8	16515	91.3	97.4
**GWD**	13695	91.8	97.3	15901	92.1	97.6
**MSL**	12676	90.7	96.9	16395	94.6	98.8
European						
**CEU**	13076	36.1	42.7	22662	74.2	82.5
**FIN**	13571	25.7	27.0	25019	71.5	80.1
**GBR**	12307	28.6	32.3	22674	71.7	80.5
**IBS**	10139	28.6	32.3	19839	59.4	70.8
**TSI**	9869	14.2	15.0	17831	49.2	60.0
Asian						
**CDX**	14204	29.8	34.0	20768	47.4	56.7
**CHB**	15447	40.3	48.9	18175	37.9	44.3
**CHS**	14499	38.9	32.7	19433	41.9	49.2
**JPT**	15539	38.4	46.8	21176	51.2	60.6
**KHV**	13010	27.3	31.2	17740	36.4	42.3
