# Supplementary material for: Population genetic analysis of the DARC locus (Duffy) reveals adaptation from standing variation associated with malaria resistance in humans
Source: PLoS Genet. 2017 Mar 10;13(3):e1006560. doi: 10.1371/journal.pgen.1006560 (PMC5365118; doi:10.1371/journal.pgen.1006560)
Supplement: S8 Table — Results for the TMRCA of FY*O major haplotype by population. Results assume 25 year generation time and mutation rate of 1.2 * 10−8 mutations per basepair per generation. Confidence intervals are calculated from 1000 bootstrapped samples. (PDF) [file pgen.1006560.s016.pdf]

**Population**	**Num. haps**	**T_{MRCA} (yrs, EHH 0.66)**	**95% CI (yrs)**	**T_{MRCA} (yrs, EHH 0.5)**	**95% CI (yrs)**
**All samples**	1229	42,183	34,100 – 49,030	40,227	33,728 – 47,043
African					
**YRI**	192	37,891	20,022 – 58,628	33,632	19,385 – 50,630
**LWK**	173	39,596	19,940 — 60,422	39,093	21,644 — 59,701
**ESN**	182	29,431	12,688 — 47,674	26,140	13,083 — 42,423
**GWD**	208	45,630	29,325 -– 65,193	40,556	24,505 — 57,283
**MSL**	149	66,749	44,256 -– 90,969	59,262	38,107 — 80,467
**Uganda**	196	41,427	16,184 — 50,124	40,682	23,437 -– 63,141
**Zulu**	102	37,417	15,312 -– 67,605	43,405	18,800 — 72,770
**Nzebi**	35	22,188	0 — 53,089	43,501	0 -– 94,791
**Baka**	33	55,151	12,780 -– 96,235	59,472	20,502 -– 98,801
**Mbuti**	7	0	0 – 0	0	0 – 0
