# Supplementary material for: Population genetic analysis of the DARC locus (Duffy) reveals adaptation from standing variation associated with malaria resistance in humans
Source: PLoS Genet. 2017 Mar 10;13(3):e1006560. doi: 10.1371/journal.pgen.1006560 (PMC5365118; doi:10.1371/journal.pgen.1006560)
Supplement: S9 Table — Results for the TMRCA of FY*O minor haplotype by population. Results assume 25 year generation time and mutation rate of 1.2 * 10−8 mutations per basepair per generation. Confidence intervals are calculated from 1000 bootstrapped samples. (PDF) [file pgen.1006560.s017.pdf]

Population	Num. haps	T_{MRCA} (yrs, EHH 0.66)	95% CI (yrs)	T_{MRCA} (yrs, EHH 0.5)	95% CI (yrs)
**All samples**	144	56,052	38,927 – 75,073	141,692	117,979 – 164,915
African					
**YRI**	21	18,395	0 – 49,668	104,514	65,321 – 132,275
**LWK**	24	82,579	0 – 120,370	167,119	86,355 – 228,034
**ESN**	14	27,593	0 – 70,044	69,718	0 – 120,593
**GWD**	17	59,650	0 – 93,735	136,166	65,561 – 173,989
**MSL**	20	57,254	0 — 112,853)	171,171	82,065 – 245,403
**Uganda**	24	108,409	66,483 — 137,420	182,440	127,358 — 209,776
**Zulu**	13	0	0 – 0	0	0 – 0
**Nzebi**	2	0	0 – 0	171,468	0 – 171,468
**Baka**	5	0	0 – 0	0	0 – 0
**Mbuti**	4	64,384	0 – 128,768	57,156	0 – 114,312
