# Supplementary material for: Population genetic analysis of the DARC locus (Duffy) reveals adaptation from standing variation associated with malaria resistance in humans
Source: PLoS Genet. 2017 Mar 10;13(3):e1006560. doi: 10.1371/journal.pgen.1006560 (PMC5365118; doi:10.1371/journal.pgen.1006560)
Supplement: S10 Table — Results for the TMRCA of FY*A by population. Results assume 25 year generation time and mutation rate of 1.2 * 10−8 mutations per basepair per generation. Confidence intervals are calculated from 1000 bootstrapped samples. (PDF) [file pgen.1006560.s018.pdf]

Population	Num. haplotypes	T_{MRCA} (years)	95% CI (years)
**All samples**	1006	57,184	47,785 – 64,732
European			
**All European**	150	67,815	43,834 – 104,192
**CEU**	32	53,993	13,060 – 96,638
**FIN**	48	91,176	36,772 – 145,788
**GBR**	18	26,309	0 – 66,812
**IBS**	20	81,225	0 – 159,969
**TSI**	32	72,393	21,305 – 121,354
Asian			
**All Asian**	856	53,046	44,506 – 61,259
**CDX**	168	72,605	51,631 – 93,332
**CHB**	180	42,377	23,576 – 61,852
**CHS**	182	39,954	24,686 – 56,254
**JPT**	164	46,269	28,099 – 64,821
**KHV**	162	62,340	42,604 – 82,663
