# Supplementary material for: Population genetic analysis of the DARC locus (Duffy) reveals adaptation from standing variation associated with malaria resistance in humans
Source: PLoS Genet. 2017 Mar 10;13(3):e1006560. doi: 10.1371/journal.pgen.1006560 (PMC5365118; doi:10.1371/journal.pgen.1006560)
Supplement: S11 Table — All nonsynonymous mutations segregating in the DARC gene region in gorillas, chimpanzees, and bonobos. (PDF) [file pgen.1006560.s019.pdf]

**Species**	**Genomic location**	**Protein change**	**Major allele (freq)**	**Minor allele (freq)**
Gorilla	Chr1:138,516,876	D42N	G (91.3%)	A (8.7%)
Chimpanzee	Chr1:137,538,083	L262R	T (94%)	G (6%)
Chimpanzee	Chr1:137,538,105	N269K	G (82%)	C (18%)
Chimpanzee	Chr1:137,538,211	A305T	G (96%)	A (4%)
Chimpanzee	Chr1:137,538,219	F307L	C (98%)	G (2%)
Bonobo	Chr1:137,537,513	G72A	G (88.5%)	C (11.5%)
Bonobo	Chr1:137,537,980	A178G	C (91%)	G (9%)
