# Supplementary material for: Population genetic analysis of the DARC locus (Duffy) reveals adaptation from standing variation associated with malaria resistance in humans
Source: PLoS Genet. 2017 Mar 10;13(3):e1006560. doi: 10.1371/journal.pgen.1006560 (PMC5365118; doi:10.1371/journal.pgen.1006560)
Supplement: S12 Table — (PDF) [file pgen.1006560.s020.pdf]

Gene	Num. sequences	Gene length (bps)	T_{MRCA} (years)
**B-tubulin**	76	670	140,789
**Crk2**	129	726	97,009
**asl**	92	838	247,388
**clpC**	66	574	71,608
**mitoD**	134	2536	141,495
**ldh**	109	771	248,678
